# Nicotine Reprograms Aging‐Related Metabolism and Protects Against Motor Decline in Mice

**DOI:** 10.1002/advs.202415311

**Published:** 2025-07-28

**Authors:** Shuhui Jia, Xiaoyuan Jing, Ruoxi Wang, Mengke Su, Pei Wang, Yingxin Feng, Xiaohu Ren, Longfang Tu, Ping Wei, Zhen Lu, Yicong Jia, Feng Hong, Zhizhun Mo, Jiemeng Zou, Kang Huang, Caiyun Yan, Qianhui Zou, Liang Wang, Guoping Zhong, Zhi Zeng, Qiuliyang Yu, Wen Su, Xifei Yang, Fan Pan, Jianzhi Wang, Liping Wang, Lijun Kang, Paul J. Kenny, Zuxin Chen, Xin‐an Liu

**Affiliations:** ^1^ Guangdong Provincial Key Laboratory of Brain Connectome and Behavior Brain Cognition and Brain Disease Institute (BCBDI) Shenzhen‐Hong Kong Institute of Brain Science Shenzhen Institutes of Advanced Technology (SIAT) Chinese Academy of Sciences (CAS) Shenzhen 518055 China; ^2^ Shenzhen Key Laboratory of Drug Addiction Shenzhen Neher Neural Plasticity Laboratory Brain Cognition and Brain Disease Institute Shenzhen‐Hong Kong Institute of Brain Science Shenzhen Institutes of Advanced Technology Chinese Academy of Sciences Shenzhen 518055 China; ^3^ Sino‐European Center of Biomedicine and Health Institute of Biomedicine and Biotechnology Shenzhen Institutes of Advanced Technology Chinese Academy of Sciences Shenzhen 518055 China; ^4^ Department of Pathology Shenzhen University Shenzhen 518055 China; ^5^ Shenzhen Key Laboratory of Modern Toxicology, Shenzhen Medical Key Discipline of Health Toxicology Shenzhen Center for Disease Control and Prevention Shenzhen 518055 China; ^6^ Centre for Cancer Immunology Shenzhen Institute of Advanced Technology Chinese Academy of Sciences Shenzhen 518055 China; ^7^ Department of Pathophysiology, Key Laboratory of Ministry of Education for Neurological Disorders, School of Basic Medicine, Tongji Medical College Huazhong University of Science and Technology Wuhan 430060 China; ^8^ Nash Family Department of Neuroscience Icahn School of Medicine at Mount Sinai New York NY 10029 USA; ^9^ Shenzhen Bayone Biotech CO., Ltd. Shenzhen 518055 China; ^10^ Research Center for Primate Neuromodulation and Neuroimaging Shenzhen Key Laboratory for Molecular Imaging Guangdong Provincial Key Laboratory of Biomedical Optical Imaging Technology Shenzhen Institutes of Advanced Technology Chinese Academy of Sciences Shenzhen 518055 China; ^11^ Institute of Clinical Pharmacology School of Pharmaceutical Sciences Sun Yat‐sen University Guangzhou 510080 China; ^12^ Department of Neurology of the Fourth Affiliated Hospital and School of Brain Science and Brain Medicine, NHC and CAMS Key Laboratory of Medical Neurobiology Zhejiang University School of Medicine Yiwu 322000 China; ^13^ Department of Pathology Renmin Hospital of Wuhan University Wuhan 430060 China

**Keywords:** aging, behavior‐metabolome age score, metabolomics, motor function, oral nicotine, sphingolipid homeostasis

## Abstract

The effects of nicotine on aging‐related motor and cognitive decline remain controversial due to limited empirical evidence. Here, mice are permitted to orally consume nicotine over a 22‐month period and observed attenuated motor decline without pathological alterations in major metabolism‐related peripheral organs or immune system dysfunction. Multi‐organ metabolomic profiling and network analysis of aged mice (24 months old) identified nicotine‐responsive pathways related to glycolipid metabolism and energy homeostasis. Dynamic gut microbiota profiling via series expression miner‐based longitudinal analysis reveals that nicotine consumption preserved microbiota composition and altered microbial‐derived metabolites associated with the sphingolipid pathway, known to regulate age‐related muscle dysfunction and sarcopenia. Assays in aged mice and C2C12 cells confirmed that nicotine regulates sphingolipid turnover, particularly via sphingomyelin synthases and neutral sphingomyelinases, to enhance nicotinamide adenine dinucleotide availability and energy metabolism. These metabolic adaptations correlated with reduced ceramide accumulation and improved motor function. Behavior‐Metabolome Age (BMAge) score confirmed a biologically younger phenotype in the nicotine‐treated mice. Together, these findings suggest that life‐long oral nicotine consumption reprograms aging‐associated metabolism through regulation of systemic sphingolipid homeostasis, conferring resilience against age‐related motor decline.

## Graphical abstract

Long‐term oral nicotine intake protects against age‐related motor decline in mice without eliciting systemic toxicity. Integrated multi‐organ metabolomic profiling and longitudinal gut microbiota analyses reveal that nicotine induces coordinated remodeling of glycolipid and sphingolipid metabolism, enhances NAD⁺ bioavailability, and suppresses ceramide accumulation, collectively supporting a systemic metabolic reprogramming that confers resilience to age‐related motor decline.

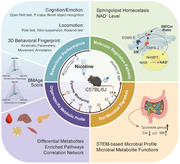

## 1 Introduction

The global aging population has intensified research into the biological mechanisms and therapeutic interventions for aging‐related functional decline. Aging is a complex, multifactorial process marked by systemic physiological deterioration, including metabolic dysregulation, reduced mobility, and hallmark features such as disrupted intercellular communication and impaired energy metabolism.^[^ ^1^ ^]^ While these manifestations exhibit organ‐specificity, their interconnectedness underscores the systemic nature of aging.^[^ ^1^, ^2^, ^3^ ^]^ Notably, mobility, cognitive function, and psychiatric health are critical determinants of healthy aging;^[^ ^4^, ^5^, ^6^ ^]^ however, the decline in physical mobility—a key component of frailty—severely compromises metabolic health.^[^ ^4^, ^7^ ^]^ Despite this, integrated models elucidating their interplay with systemic metabolism remain scarce.

Recent advances in aging research have employed multidimensional omics and machine learning–based approaches to characterize biological aging trajectories.^[^ ^8^, ^9^ ^]^ These studies have revealed coordinated changes in energy metabolism, mitochondrial efficiency, redox balance, and nutrient signaling networks across tissues. Among metabolic hallmarks of aging, the decline in nicotinamide adenine dinucleotide (NAD⁺) levels impair cellular energy homeostasis and genomic integrity, while its restoration can mitigate age‐related decline.^[^ ^10^ ^]^ Likewise, sphingolipid pathway dysregulation—marked by ceramide accumulation—contributes to sarcopenia and motor dysfunction in aging.^[^ ^11^ ^]^ These highlight metabolism as a therapeutic axis for healthy aging. While these metabolic factors collectively underpin novel anti‐aging strategies, the influence of environmental variables (e.g., nicotine) on aging trajectories remains poorly defined.

The global tobacco epidemic persists as a major public health threat, with nicotine—the principal psychoactive compound in tobacco—demonstrating paradoxical effects on aging and age‐related diseases.^[^ ^12^, ^13^ ^]^ On one hand, chronic smoking is associated with increased risks for cancer,^[^ ^14^ ^]^ metabolic syndrome,^[^ ^15^ ^]^ and premature mortality.^[^ ^16^ ^]^ On the other hand, epidemiological data suggest protective associations between smoking and certain conditions^[^ ^17^ ^]^ such as ulcerative colitis,^[^ ^18^ ^]^ Parkinson's disease,^[^ ^19^ ^]^ specific subtypes of Alzheimer's disease,^[^ ^20^ ^]^ and type I diabetes.^[^ ^21^ ^]^ These divergent outcomes suggest that nicotine per se, rather than tobacco smoke as a whole, may possess distinct and context‐dependent biological activities. Recent studies have shown that low‐dose nicotine enhances NAD⁺ biosynthesis and improves metabolic resilience, suggesting potential anti‐aging benefits.^[^ ^22^ ^]^ These beneficial effects occur at nicotine concentrations significantly lower than those experienced through smoking, emphasizing the need for dose‐ and duration‐specific investigations.^[^ ^23^ ^]^

Concurrently, the gut microbiota has gained recognition as a key regulator of systemic physiology, integrating metabolic, immune, and neurological functions.^[^ ^24^, ^25^, ^26^ ^]^ Aging drives significant alterations in microbial composition,^[^ ^27^, ^28^ ^]^ marked by a reduction in beneficial commensals (e.g., *Bifidobacterium*, *Lactobacillus*, *Faecalibacterium*, *Eubacterium*) and enrichment of pro‐inflammatory or opportunistic taxa (e.g., *Proteobacteria*, *Eggerthella*, *Alistipes*). In contrast, healthy aging—exemplified by centenarians—is often associated with the retention or enrichment of butyrate‐producing species such as *Roseburia* and *Akkermansia muciniphila*, which promote metabolic homeostasis.^[^ ^29^ ^]^ Nicotine's impact on gut microbiota varies by administration route (e.g., oral vs subcutaneous), exposure duration, and host factors like age and metabolic status.^[^ ^30^, ^31^, ^32^, ^33^ ^]^ While typically increasing *Actinobacteria* while reducing *Bacteroidetes*,^[^ ^34^ ^]^ these effects may differ across conditions, highlighting the need for context‐specific analysis of nicotine‐gut microbiome interactions.

In this study, we systematically investigated the long‐term effects of oral nicotine administration on aging in mice, assessing cognitive, psychiatric, and motor functions at 14, 18, 22, and 24 months of age under low (0.25 g L^−1^, 0.25Nico) and high (0.5 g L^−1^, 0.5Nico) nicotine exposure. Metabolomic profiling was performed in 24‐month‐old mice across key metabolic organs including the liver, white adipose tissue (WAT), pancreas, and muscle, alongside plasma. To model voluntary nicotine intake while minimizing aversive effects, nicotine—known for its bitter taste and low pH—was co‐administered with sodium saccharin. The study also utilized the Behavior‐Metabolome Age (BMAge) Score system to integrate behavioral phenotypes with metabolic signatures and provide a composite assessment of nicotine's impact on systemic aging.^[^ ^35^ ^]^ This integrative approach allowed us to elucidate how long‐term nicotine exposure influences behavioral aging and systemic metabolic remodeling, potentially through mechanisms involving the gut–brain–metabolism axis.

## 2 Results

### 2.1 Lifelong Oral Nicotine Administration Attenuates Age‐Related Motor Decline

**Figure 1:**
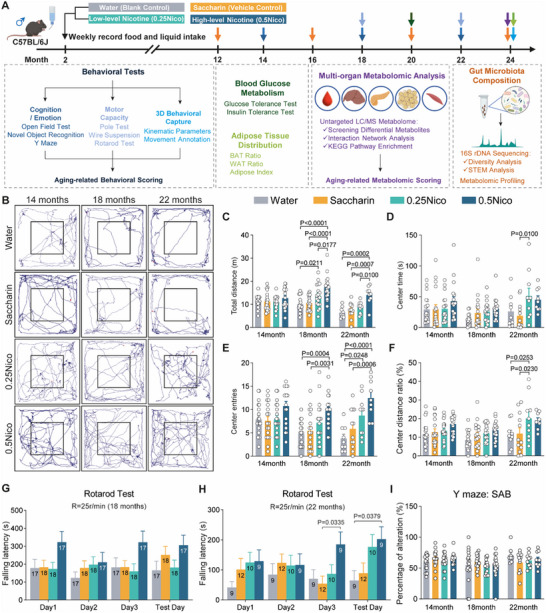
Long‐term oral nicotine treatment attenuates age‐related motor decline in mice. A) Experimental timeline. Four groups of mice received treatment from 2 to 24 months of age. Weekly monitoring of food and liquid intake was performed. Fecal samples were collected for sequencing at 12, 16, 20, and 24 months. Behavioral assessments were conducted at 14, 18, 22, and 24 months, while GTT and ITT tests were performed at 20 months. At 24 months, mice were euthanized for tissue collection and adipose tissue distribution analysis. Time points for each procedure are indicated by arrows (color‐coded by assay type). B–F) OFT performance. Representative tracing plots B), total distance travelled C), time spent in the center zone D), number of center entries E), and ratio of center‐to‐total distance traveled F) from the OFT across different groups. G–H, Rotarod endurance assessment. Falling latency at 18 months (10‐min test, 25 rpm) G) and 22 months (5‐min test, 25 rpm) H). The sample size N) for each group is indicated on the corresponding bar. I, Frequency of SAB in the Y‐maze among the groups. Data in C–I were analyzed by two‐way ANOVA with Tukey's post hoc test. Values represent mean ± SEM; group sizes (n = 9–20) are indicated on bars. GTT, glucose tolerance test; ITT, insulin tolerance test; OFT, Open field test; SAB, spontaneous alternation behavior.

To investigate the effects of lifelong oral nicotine exposure on aging, male C57BL/6J mice were provided with nicotine in their drinking water at concentrations of 0.25 g L^−1^ (0.25Nico, low dose) and 0.5 g L^−1^ (0.5Nico, high dose), starting at 8 weeks of age and continuing for 22 months. Behavioral assessments—including evaluations of motor and non‐motor functions—were performed at 14, 18, 22, and 24 months to examine spontaneous activity, cognitive function, psychiatric‐like behaviors, and motor performance (**Figure** **1**A). Liquid and food intake were monitored throughout the study. Notably, mice in the 0.25Nico group consumed significantly more liquid compared to the other groups (F _(3, 180)_ = 37.65, P _Treatment_ < 0.0001; all P < 0.0001 for 0.25Nico vs Water, Saccharin, and 0.5Nico), with the area under the curve (AUC) also significantly higher than that of the Water (P = 0.0272) and 0.5Nico groups (P = 0.0157). Food intake remained consistent across all groups, suggesting a selective preference for the 0.25Nico solution (Figure S1A,B, Supporting Information). The efficacy of oral nicotine delivery was confirmed by liquid chromatography–mass spectrometry (LC‐MS) analysis, which detected both nicotine and its primary metabolite, cotinine, in treated mice, thereby validating successful systemic exposure (Figure S1C, Supporting Information). Although the 0.25Nico group exhibited greater fluid intake, LC‐MS results revealed higher plasma nicotine concentrations in the 0.5Nico group. This indicates that systemic nicotine levels are influenced by both the volume of fluid consumed and the nicotine concentration in the solution.

Aging is closely associated with declines in motor function, cognitive performance, and increased susceptibility to mood disorders.^[^ ^36^, ^37^ ^]^ To comprehensively assess these domains, mice underwent a behavioral test battery—including the open field test (OFT), pole test, wire suspension test, rotarod test, novel object recognition (NOR) test, and Y‐maze—at 16, 18, and 22 months of age. Notably, nicotine administration, particularly at the 0.5 g L^−1^ dose (0.5Nico), significantly enhanced spontaneous locomotor activity and reduced anxiety‐like behaviors at 18 and 22 months (Figure 1B–F). In the OFT, nicotine treatment increased total distance travelled (Total distance: F_(2, 176)_ = 12.81, P _Time_ < 0.0001, F_(3, 176)_ = 18.17, P _Nicotine_ < 0.0001), with the 0.5Nico group showing significantly higher activity than Water (P < 0.0001), Saccharin (P < 0.0001), and 0.25Nico (P = 0.0177) groups at 18 months, and compared to Water (P = 0.0002), Saccharin (P = 0.0007), and 0.25Nico (P = 0.01) at 22 months (Figure 1C). Additionally, nicotine increased center time (F _(3, 176)_ = 5.242, P _Nicotine_ = 0.0017, Figure 1D), number of center entries (F _(2, 176)_ = 4.023, P _Time_ = 0.0196, F _(3, 176)_ = 17.61, P _Nicotine_ < 0.0001, Figure 1E), and center distance ratio (F _(2, 176)_ = 5.75, P _Time_ = 0.0038, F_(3, 176)_ = 7.68, P_Nicotine_ < 0.0001, Figure 1F), indicating improved locomotor activity and reduced anxiety‐like behavior.

While the pole test showed no significant differences among groups at 18 months (Figure S2A, Supporting Information), significant effects of both training (F _(3,107)_ = 3.832, P = 0.0119) and treatment group (F _(3,107)_ = 4.321, P = 0.0064) emerged at 22 months, with notable improvement observed in the 0.5Nico group compared to Saccharin (P = 0.0053; Figure S2B, Supporting Information), indicating a protective effect of nicotine on motor coordination. The wire suspension test (Figure S2C,D, Supporting Information) further supported enhanced motor strength and endurance in the 0.5Nico group, particularly at 18 months (F _(2.75,186.1)_ = 4.472, P = 0.006; F _(3,68)_ = 4.741, P = 0.0046). Similarly, in the rotarod test, 0.5Nico‐treated mice exhibited significantly longer latency to fall at 25 rpm at 18 months (10 rpm: F_(2.671,172.7)_ = 74.53, P _Training_ < 0.0001, Figure S2F (Supporting Information); 15 rpm: F_(2.752,177.9)_ = 42.77, P _Training_ < 0.0001, F_(9,194)_ = 2.096, P _Interaction_ = 0.0316, Figure S2G (Supporting Information); 20 rpm: F_(2.473,159.9)_ = 15.85, P _Training_ < 0.0001, Figure S2H (Supporting Information); 25 rpm: F_(3,264)_ = 5.864, P _Training_ = 0.0007, P_0.5Nico‐Water_ = 0.001, P_0.5Nico‐Saccharin_ = 0.0324, P_0.5Nico‐0.25Nico_ = 0.0044, Figure 1G). No significant differences were observed at higher speeds (30 and 40 rpm; Figure S2I–J, Supporting Information). At 22 months, although the overall training effect was not significant, 0.5Nico‐treated mice consistently outperformed other groups at moderate speeds (15–25 rpm) (15 rpm: F_(3,144)_ = 6.557, P _Nicotine_ = 0.0003, P_0.5Nico‐Water_ = 0.0091, Figure S2K (Supporting Information); 20 rpm: F_(3,144)_ = 6.557, P _Nicotine_ = 0.0003, P0.5Nico‐Water = 0.0001, P_0.5Nico‐Saccharin_ = 0.0377, P_0.25Nico‐Water_ = 0.0447, Figure S2L (Supporting Information); 25 rpm: F_(3,144)_ = 5.183, P _Nicotine_ = 0.002, P_0.5Nico‐Water_ = 0.002, P_0.5Nico‐Saccharin_ = 0.0306, Figure 1H). These results suggest that long‐term oral nicotine administration enhances motor coordination and endurance during aging.

Cognitive assessments using the NOR and Y‐maze revealed significant effects of time (NOR: F _(1.660,143.6)_ = 5.409, P = 0.0086; Y‐maze: F _(1.998,177.0)_ = 5.896, P = 0.0034), reflecting age‐related cognitive decline. However, no significant effects of nicotine treatment on memory performance were observed (Figure S2E, Supporting Information, Figure 1I), suggesting that cognitive deterioration occurred independently of nicotine exposure.

Correlation analyses at 18 months revealed modest negative associations between cognitive and anxiety‐related metrics—for example, a slight inverse correlation between center distance ratio and Y‐maze performance (r = –0.079, P = 0.025). In contrast, positive correlations emerged between anxiety‐like behavior and motor performance, such as between center time in the open field and rotarod latency at 15 rpm (r = 0.219, P = 0.013) and 40 rpm (r = 0.291, P = 0.014; Figure S2M, Supporting Information). By 22 months, motor‐related measures remained positively correlated (e.g., center time vs rotarod at 25 rpm: r = 0.387, P = 0.014; center distance ratio: r = 0.339, P = 0.032; Figure S2N, Supporting Information), while anxiety‐related metrics displayed inverse relationships with motor performance. Taken together, these findings indicate that long‐term oral nicotine administration—particularly at the 0.5 g L^−1^ dose—enhanced motor function and reduced anxiety‐like behavior in aged mice, without significantly impacting age‐associated cognitive decline.

**Figure 2:**
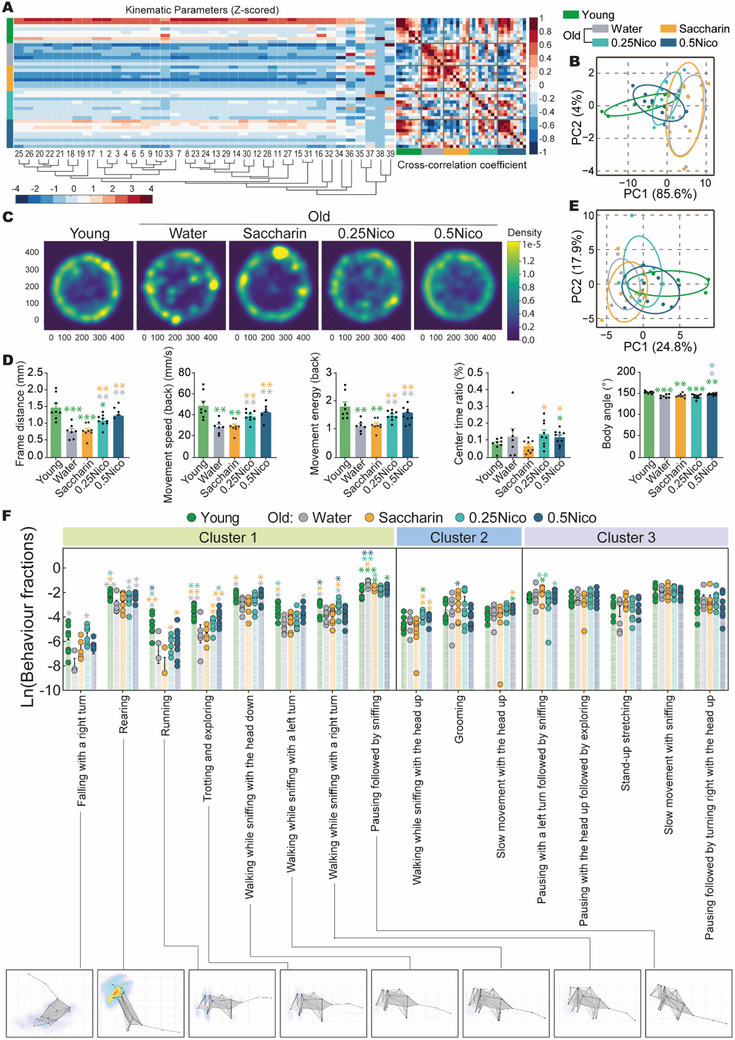
Oral nicotine modulates spontaneous behavioral kinematics in mice. A) Hierarchical clustering and correlation analysis of kinematic parameters. Complete linkage clustering and Pearson's correlation heatmap of 39 kinematic parameters across experimental groups, derived from a 3D motion‐capture learning framework. B) PCA plot illustrating 39 kinematic parameters across the groups. C) Enriched heatmap showing stopping duration across groups, as quantified by 3D behavioral tracking. D) Representative differential kinematic parameters from the 3D‐behavioral analysis, exhibiting significant group differences: frame distance, movement speed (back), movement energy (back), center time ratio, and body length. E) PCA plot showing 40 distinct movement sequences across different groups. F) Representative 3D behavioral trajectories grouped by functional clusters: Cluster 1: behaviors modulated by both aging and nicotine administration; Cluster 2: behaviors specifically altered by saccharin or nicotine consumption; Cluster 3: behaviors showing no significant differences across groups. The Welch's *t*‐test was applied in panels D and F. Data are presented as mean ± s.e.m., with n = 7–9 in each group; *p < 0.05, **p < 0.01, ***p < 0.001. PCA, Principal component analysis.

To investigate behavioral and postural changes in aged mice, 3D behavioral capture and analysis^[^ ^38^ ^]^ were conducted at 24 months of age, with 8‐month‐old water‐treated mice serving as the Young control group. AI‐based analysis revealed that chronic nicotine administration modulated various kinematic parameters (see Table S1, Supporting Information), with the 0.5Nico group exhibiting behavioral profiles more closely resembling those of young mice compared to other groups (**Figure** **2**A,B). Representative track plots from the OFT demonstrated that both the 0.25Nico and 0.5Nico groups spent less time stationary than the Water and Saccharin groups (Figure 2C). A comprehensive summary of behavioral parameters and unsupervised manifold projections (UMAP) using support vector machine (SVM) classification across all groups is provided in Figure S3 and Table S1 (Supporting Information).

Consistent with prior locomotor assessments, motor‐related metrics were significantly enhanced in nicotine‐treated mice. Compared to the 0.25Nico group, Saccharin‐treated mice exhibited significantly reduced frame distance (P = 0.0038), movement speed (back) (P = 0.003), and movement energy (back) (P = 0.0024). Similarly, compared to the 0.5Nico group, Saccharin mice displayed further reductions in these motor parameters (P _Frame distance_ = 0.0023, P _Movement speed (back)_ = 0.0019, and P _Movement energy (back)_ = 0.0022). Anxiety‐related metrics also revealed significant differences: the center time ratio was increased in both the 0.25Nico (P = 0.0269) and 0.5Nico (P = 0.0141) groups relative to Saccharin. Furthermore, the 0.5Nico group showed greater body angle stretch compared to 0.25Nico (P = 0.0094; Figure 2D; Table S1, Supporting Information), indicative of enhanced exploratory engagement. An unsupervised deep learning model identified 40 distinct spontaneous behaviors, which were clustered into 16 behavioral sequences based on clustering algorithms and manual pattern validation (Table S2, Supporting Information). Notable group differences were observed across multiple sequences (Figure S4A,C,E,G,I; Table S2, Supporting Information). Postural dynamics clearly distinguished nicotine‐treated from vehicle‐treated groups (Figure S4F,H, Supporting Information), with nicotine‐treated mice displaying patterns more similar to young controls (Figure 2E), suggesting a dose‐dependent induction of specific behavioral signatures by nicotine (Figure S4J, Supporting Information). Seven behavior sequences—falling with a right turn, rearing, running, trotting and exploring, walking while sniffing with the head down, walking while sniffing with a left turn, and walking while sniffing with a right turn—were significantly diminished with age but were robustly restored in both the 0.25Nico and 0.5Nico groups. In contrast, the frequency of pausing followed by sniffing increased with age and was not affected by nicotine treatment (Figure 2F; Figure S4A,E,G; Table S2, Supporting Information). In addition, behaviors potentially related to nicotine's psychoactive effects, such as grooming, slow movement with the head up, and walking while sniffing with the head up, were also identified and may serve as markers of nicotine‐induced behavioral phenotypes (Figure 2F; Table S2, Supporting Information). Overall, these findings demonstrate that aging leads to significant alterations in motor and exploratory behaviors as well as spontaneous movement sequences. While Saccharin showed minimal influence on age‐related behavioral changes, nicotine—particularly at 0.5 g L^−1^—attenuated age‐associated behavioral decline. Importantly, it preserved complex, learned exploratory behaviors and induced distinct, nicotine‐associated behavioral signatures. Collectively, the data indicate that long‐term oral nicotine use protects against a senescent behavioral profile in aged mice.

### 2.2 Lifelong Oral Nicotine Administration does not Impair Basal Metabolism

Based on the observed improvement in motor function, we next sought to investigate the effects of nicotine on basal metabolism in aged mice. Given previous reports highlighting nicotine's influence on glycemic regulation,^[^ ^15^, ^39^, ^40^ ^]^ we performed glucose tolerance tests (GTT) and insulin tolerance tests (ITT) in 20‐month‐old mice. These assessments revealed no significant differences between vehicle‐ and nicotine‐treated groups (Figure S1D,E, Supporting Information). However, at the time of sacrifice (24 months of age), altered adipose tissue distribution was noted despite no significant differences in overall body weight. Specifically, the visceral‐to‐subcutaneous fat (VF/SF) ratio was significantly elevated in the 0.5Nico group compared to the Water, Saccharin, and 0.25Nico groups. Although total visceral fat content—including perirenal and epididymal fat (EF)—was comparable across groups, this shift in fat distribution suggests increased accumulation of WAT in the celiac visceral region in the 0.5Nico group. SF, brown adipose tissue (BAT), gastrocnemius muscle mass, and overall adiposity index remained unchanged (Figure S1F, Supporting Information). In contrast to previous findings involving smoked, subcutaneous, or intraperitoneal nicotine administration,^[^ ^15^, ^39^, ^40^ ^]^ long‐term oral nicotine intake did not result in hyperglycemia in our model. These results indicate that while chronic oral nicotine may alter fat distribution patterns, it does not adversely affect systemic glucose metabolism in aged mice.

To assess potential organ toxicity associated with long‐term nicotine consumption, histopathological examinations were performed on the liver, pancreas, WAT, and skeletal muscle in both the Saccharin and 0.5Nico groups (Figure S1G, Supporting Information). Liver analysis revealed mild hyaline degeneration and focal inflammatory infiltration in both groups, consistent with age‐related spontaneous pathological changes commonly observed in mice and not attributable to nicotine exposure. Similarly, mild autophagic vesicles were detected in pancreatic acinar cells across all groups, without accompanying inflammatory infiltration or structural abnormalities in WAT or muscle tissue. These findings suggest that chronic oral nicotine administration does not induce detectable organ toxicity in aged mice. In parallel, flow cytometry analysis of immune function revealed no significant differences in peripheral immune responses between the 0.5Nico and Saccharin groups (Figure S1H; Figure S5, Supporting Information), further supporting the safety of long‐term oral nicotine exposure under the conditions tested.

### 2.3 Multi‐Organ Metabolomic Profiling in Aged Mice Following Oral Nicotine Administration

Given the close association between motor function, energy expenditure, and systemic metabolism,^[^ ^41^, ^42^, ^43^ ^]^ we conducted untargeted metabolomic profiling (LC‐MS) across multiple tissues—including plasma, liver, pancreas, WAT, and skeletal muscle—in 24‐month‐old mice following nicotine administration. For comparison, tissues from 8‐month‐old water‐treated mice served as the Young control group. Since 0.2% saccharin sodium was used as the vehicle control, we first assessed its metabolic impact to exclude potential confounding effects that might obscure nicotine‐specific responses.

Our results showed that lifelong consumption of 0.2% saccharin from adulthood induced minimal metabolic alterations compared to the Water group. This effect was particularly modest when contrasted with the pronounced metabolic differences observed between young and aged mice. Volcano plots revealed that although the saccharin group exhibited some changes in metabolite abundance across several organs relative to the Water group, the most prominent differences were observed in plasma, while the pancreas showed the least variation (Figure S6A–E; Table S3, Supporting Information). Correspondingly, bubble plots of KEGG pathway enrichment based on these differential metabolites further confirmed limited saccharin‐induced metabolic modulation across tissues (Figure S6F–J; Table S3, Supporting Information). Together, these findings indicate that long‐term intake of 0.2% saccharin has negligible effects on systemic metabolism in aged mice, supporting its suitability as a vehicle control in this study.

### 2.4 Multi‐Organ Metabolic Responses to Long‐Term Oral Nicotine Intake in Aged Mice

**Figure 3:**
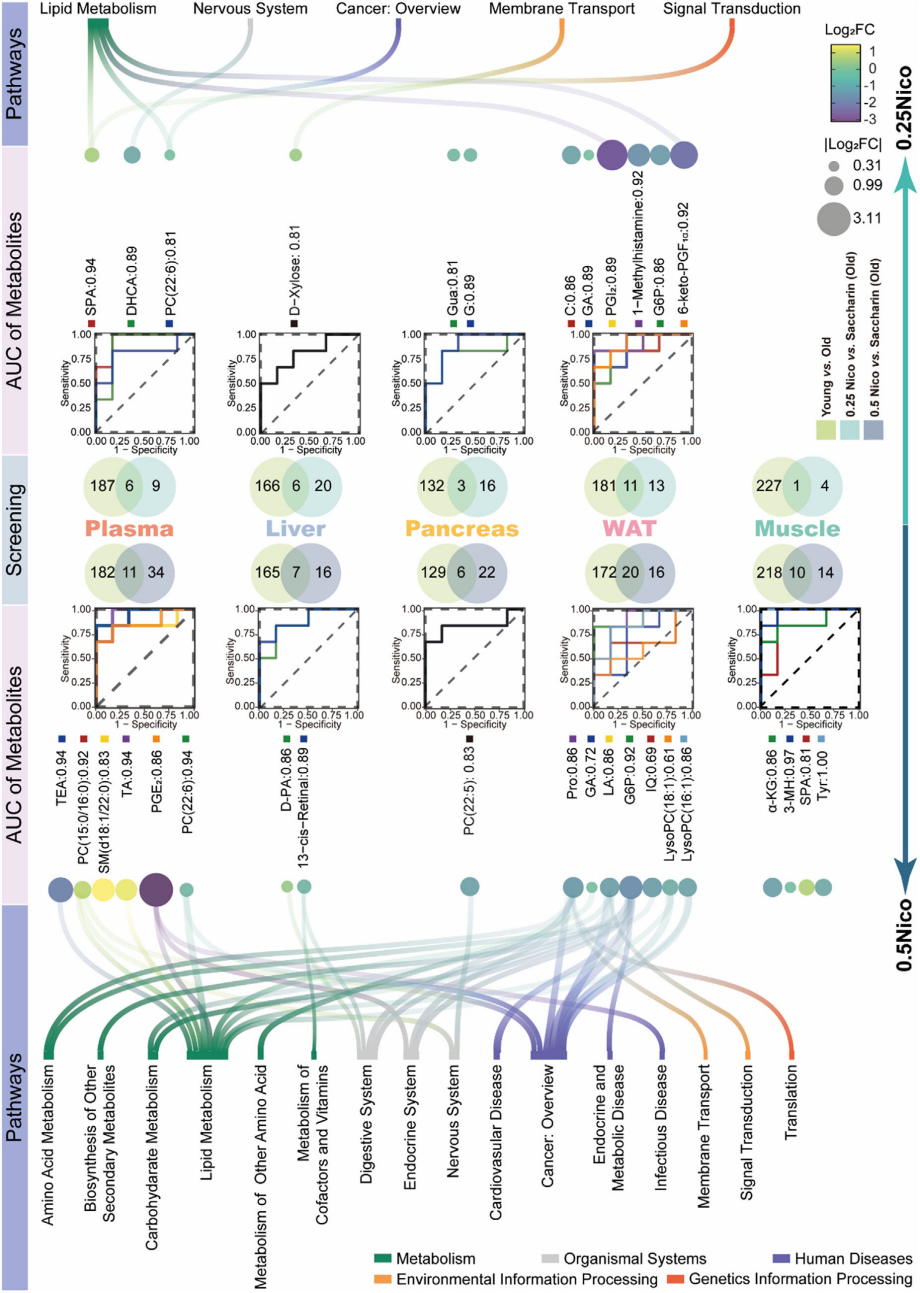
Nicotine reprograms the metabolomic profiling of multiple organs in aged mice. The differential metabolites regulated by 0.25Nico and 0.5Nico in multiple organs, including the plasma, liver, pancreas, WAT and muscle of aged mice were screened and presented in the Venn diagrams. ROC curve analysis with AUC values identifying the most robust metabolic biomarkers. Bubble plots illustrate fold‐change differences of significant metabolites relative to the Saccharin group. The significantly enriched KEGG L2 pathways were presented. WAT, white adipose tissue; ROC, Receiver operating characteristic; AUC, area under the curve. SPA, Sphinganine; DHCA, 3,4−Dihydroxyhydrocinnamic acid; Gua, Guanosine; G, Guanine; C, Cytidine; GA, Glycolic acid; PGI_2_, Prostaglandin I2; G6P, Glucose 6‐phosphate; 6‐keto‐PGF_1α_, 6‐keto‐prostaglandin F1α; TEA, Triethanolamine; TA, Traumatic acid; PGE_2_, Prostaglandin E2; PC(22:6), PC(22:6(4Z,7Z,10Z,13Z,16Z,19Z)/18:1(11Z)); D‐PA, D‐pantothenic acid; PC(22:5), PC(22:5(7Z,10Z,13Z,16Z,19Z)/18:1(11Z)); Pro, L−Proline; LA, L‐Lactic acid; IQ, Indole‐5,6‐quinone; LysoPC(18:1), LysoPC(18:1(9Z)); LysoPC(16:1), LysoPC (16:1(9Z)/0:0); α‐KG, oxoglutaric acid; 3‐MH, 3‐methylhistidine; Tyr, L‐Tyrosine.

To further investigate the metabolic effects of oral nicotine administration, we performed untargeted metabolomic analyses in aged mice treated with 0.25 g L^−1^ (0.25Nico) and 0.5 g L^−1^ (0.5Nico) nicotine across multiple organs. Principal coordinate analysis (PCA) revealed distinct metabolic shifts in both nicotine‐treated groups compared to controls (Figure S7A–E, Supporting Information). An overview of the metabolomic profiles across organs under nicotine treatment is presented in **Figure** **3**. Volcano plots illustrate significant differences in metabolite abundance in comparisons between Young and Old, 0.25Nico and Saccharin, and 0.5Nico and Saccharin across various tissues. Among these, skeletal muscle showed the highest number of differential metabolites in the Young versus Old comparison, whereas the pancreas exhibited the fewest. In the 0.25Nico group, the most pronounced metabolic changes occurred in plasma, with minimal changes in muscle. Conversely, the 0.5Nico group demonstrated the strongest effects in plasma and the least in the liver. To ensure that observed differences were nicotine‐specific, metabolites previously found to be affected by saccharin were excluded from the comparisons between nicotine and Saccharin groups. Shared differential metabolites were identified between the Young versus Old and 0.25Nico versus Saccharin or 0.5Nico versus Saccharin comparisons, many of which exhibited consistent directions of change (up‐ or downregulation), suggesting that both nicotine doses may partially reverse age‐associated metabolic alterations (Figure S8 Tables S4 and S5, Supporting Information). For example, in the liver, D‐xylose was significantly upregulated in both the 0.25Nico and Young groups (six overlapping metabolites in total). In contrast, one metabolite in skeletal muscle showed opposite changes between the 0.25Nico and Young groups. In the pancreas, guanosine (Gua) and guanine (G) were significantly downregulated in both the 0.25Nico and Young groups (three shared metabolites). In plasma, 3,4‐dihydroxyhydrocinnamic acid (DHCA) and phosphatidylcholine (PC(22:6(4Z,7Z,10Z,13Z,16Z,19Z)/18:1(11Z))) (PC(22:6)) were downregulated, while sphinganine (SPA) was upregulated in both groups (six shared metabolites). Additional plasma metabolites, including glucose 6‐phosphate (G6P), glycolic acid (GA), cytidine (C), prostaglandin I_2_ (PGI_2_), 1‐methylhistamine (MHA), and 6‐keto‐prostaglandin F1α (6‐keto‐PGF_1α_), were also significantly upregulated in both the 0.25Nico and Young groups (11 metabolites in total). KEGG pathway enrichment analysis of these shared differential metabolites—exhibiting parallel trends in the 0.25Nico and Young groups—is shown in Figure S7F and detailed in Table S4 (Supporting Information).

In the 0.5Nico group, 13‐cis‐retinal was significantly upregulated and D‐pantothenic acid (D‐PA) was downregulated in the liver, mirroring changes observed in the Young group (seven overlapping metabolites). In skeletal muscle, oxoglutaric acid (α‐KG), 3‐methylhistidine (3‐MH), and L‐tyrosine (Tyr) were downregulated, while SPA was upregulated in both the 0.5Nico and Young groups (ten shared metabolites). Similarly, PC(22:5(7Z,10Z,13Z,16Z,19Z)/18:1(11Z)) (PC(22:5)) was downregulated in the pancreas in both groups (six overlapping metabolites). In plasma, metabolites such as DHCA, PC(22:6), triethanolamine (TEA), and prostaglandin E2 (PGE_2_) were downregulated, whereas PC(15:0/16:0), sphingomyelin SM(d18:1/22:0), and traumatic acid (TA) were upregulated in both the 0.5Nico and Young groups (eleven shared metabolites). In WAT, a total of 20 metabolites—including G6P, GA, indole‐5,6‐quinone (IQ), L‐lactic acid (LA), L‐proline (Pro), LysoPC(18:1(9Z)) (LysoPC(18:1)), and LysoPC(16:1(9Z)/0:0) (LysoPC(16:1))—were significantly downregulated in both the 0.5Nico and Young groups. KEGG pathway enrichment analysis of these commonly regulated metabolites revealed strong consistency with metabolic trends observed in young mice, as shown in Figure S7G and detailed in Table S5 (Supporting Information). These results suggest that oral administration of 0.5 g L^−1^ nicotine may partially restore a youthful metabolic profile across multiple organs in aged mice.

**Figure 4:**
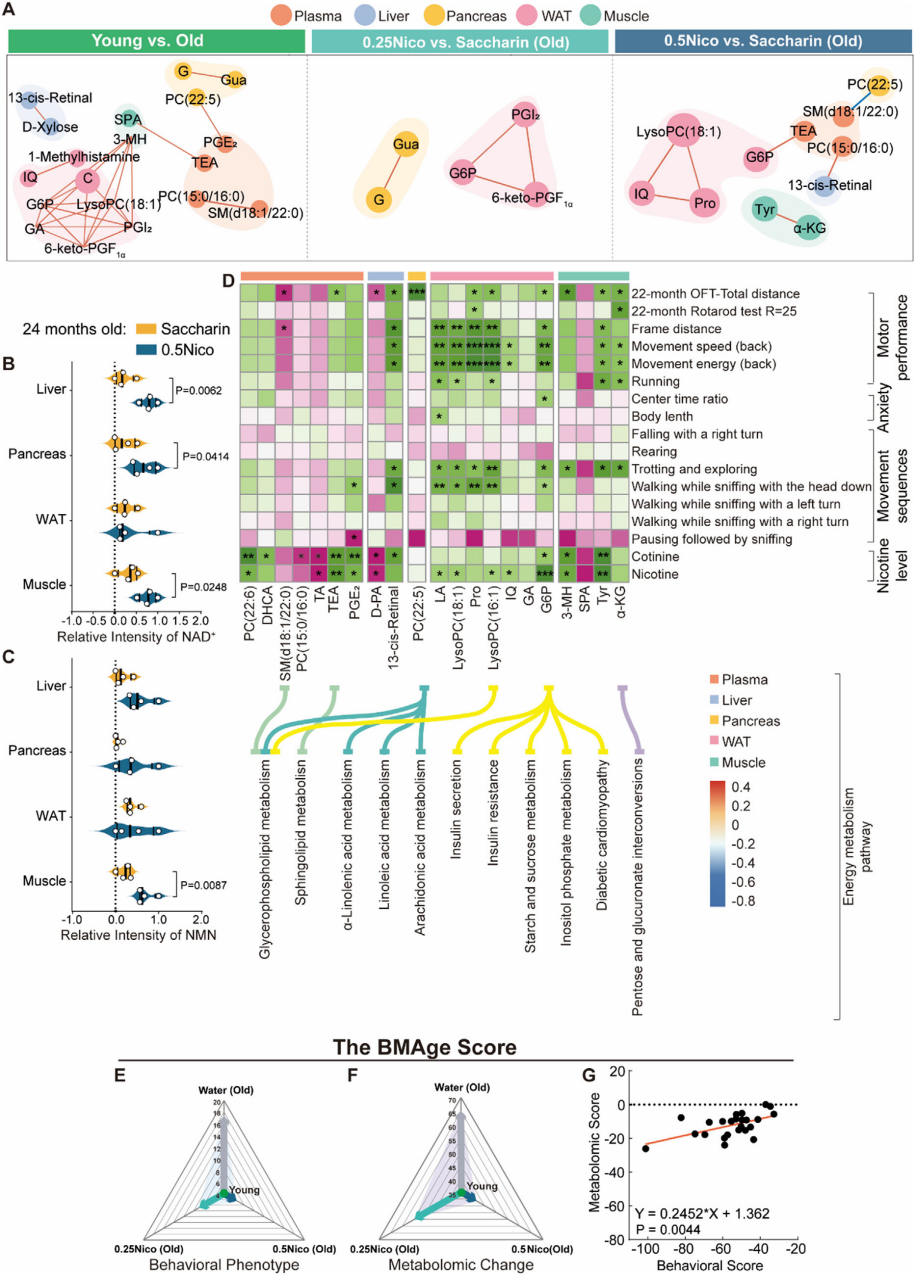
Long‐term oral nicotine administration regulates the level of metabolites related to energy metabolism in aged mice. A) Undirected metabolic network of differential metabolites across multiple organs comparing Old versus Young, 0.25 mg mL^−1^ nicotine (0.25Nico) versus Saccharin, and 0.5 mg mL^−1^ nicotine (0.5Nico) versus Saccharin groups, generated using CoSE layout (|Correlation| > 0.8, *P* < 0.05). B,C) Tissue levels of NAD⁺ (B) and nicotinamide mononucleotide (NMN) (C) in the liver, pancreas, white adipose tissue (WAT), and muscle were compared between the Saccharin and 0.5Nico groups. D) Heatmap showing correlations between differential metabolites and phenotypic features in the 0.5Nico group across multiple organs, with enrichment in energy metabolism‐related pathways. E,F) Integrated Behavior‐Metabolome Age (BMAge) scores in aged mice following long‐term oral nicotine administration. Z‐score radar plots illustrate aging‐associated behavioral phenotypes (E) and metabolomic alterations (F). G) Simple linear regression analysis between behavioral and metabolomic BMAge scores in aged mice. Mice received nicotine for a duration of 22 months. Data are presented as mean ± s.e.m., n = 6 per group. Statistical analysis in panels B–C was performed using Student's *t*‐test. *p < 0.05, **p < 0.01, ***p < 0.001. CoSE, compound spring embedder. G, Guanine; Gua, Guanosine; PC(22:5), PC(22:5(7Z,10Z,13Z,16Z,19Z)/18:1(11Z)); PGE_2_, Prostaglandin E2; TEA, Triethanolamine; PC(22:6), PC(22:6(4Z,7Z,10Z,13Z,16Z,19Z)/18:1(11Z)); IQ, Indole‐5,6‐quinone; C, Cytidine; G6P, Glucose 6‐phosphate; LysoPC(18:1), LysoPC(18:1(9Z)); GA, Glycolic acid; PGI_2_, Prostaglandin I2; 6‐keto‐PGF_1α_, 6‐keto‐prostaglandin F1α; Pro, L−Proline; DHCA, 3,4−Dihydroxyhydrocinnamic acid; TA, Traumatic acid; D‐PA, D‐pantothenic acid; LA, L‐Lactic acid; LysoPC(16:1), LysoPC (16:1(9Z)/0:0); 3‐MH, 3‐methylhistidine; SPA, Sphinganine; Tyr, L‐Tyrosine; α‐KG, oxoglutaric acid.

Compound spring embedder (CoSE) layout analysis of the multi‐organ, undirected metabolic network in the 0.25Nico and 0.5Nico groups identified WAT as a central hub in age‐related metabolic regulation, suggesting it plays a key role in mediating the beneficial metabolic effects of nicotine. Differential metabolites in the liver were predominantly enriched in pathways related to membrane transport, digestion, cofactor metabolism, and vitamin metabolism. In the pancreas, altered metabolites were significantly associated with nervous system pathways. Plasma metabolites showed enrichment in pathways related to the nervous system, lipid metabolism, cancer (overview), digestion, and parasitic infectious diseases. In WAT, metabolic changes were enriched in pathways involving digestion, endocrine function, lipid and carbohydrate metabolism, amino acid metabolism, and cancer‐related processes (**Figure** **4**A,D).

To identify potential biomarkers associated with nicotine's metabolic effects, receiver operating characteristic (ROC) curve analysis was conducted, with a threshold area under the curve (AUC) > 0.85 used for selection. Chronic administration of 0.25Nico resulted in nine potential biomarkers, including G (in pancreas), C, GA, G6P, prostaglandin (PG), MHA, 6‐keto‐PGF_1α_ (in WAT), SPA, and DHCA (in plasma). In contrast, 0.5Nico consumption yielded 13 candidate biomarkers in aged mice: D‐PA and 13‐cis‐retinal (in liver), α‐KG and 3‐MH (in muscle), PC(22:6) (in plasma), TEA, PC(15:0/16:0), TA, PGE_2_, G6P, LA, Pro, and LysoPC(16:1) (in WAT) (Figure 3). These findings underscore nicotine's dose‐dependent influence on tissue‐specific metabolism and highlight potential metabolic biomarkers associated with its long‐term effects in aged mice.

### 2.5 Key Metabolomic Signatures Regulated by Oral Nicotine Intake

As demonstrated by the preceding analyses—particularly those focused on energy‐related metabolites—nicotine intake appears to confer protective effects against motor decline in aged mice through modulation of metabolic pathways. In the 0.25Nico group compared to the Saccharin group, L‐leucine (L‐Leu) was significantly upregulated in both the liver and pancreas, while G6P and GA were downregulated in WAT, alongside Gua in the pancreas and GA in plasma (Figure S7H, Supporting Information). In the 0.5Nico versus Saccharin comparison, L‐lysine (Lys) in plasma and L‐Leu in the pancreas were upregulated. Meanwhile, GA, G6P, and LA in WAT; Tyr and α‐KG in muscle; and itaconic acid (IA) in the liver were significantly downregulated (Figure S7I, Supporting Information).

To further validate the impact of nicotine on energy metabolism during aging, levels of NAD⁺ and β‐nicotinamide mononucleotide (NMN) were measured. Results revealed significant elevations in the 0.5Nico group, indicating enhanced energy metabolism under chronic nicotine exposure (Liver: P_NAD+_ = 0.0062; Muscle: P_NAD+_ = 0.0248, P_NMN_ = 0.0087; 0.5Nico vs Saccharin, two‐tailed unpaired t‐test; Figure 4B,C). These findings suggest that long‐term oral nicotine intake modulates key energy‐related metabolites and pathways, potentially supporting metabolic resilience and motor function in aging.

### 2.6 Locomotion‐Associated Metabolites Altered by Oral Nicotine in Aged Mice

To further elucidate the metabolomic alterations induced by oral nicotine, pathway analysis was conducted to identify key differential metabolites in the 0.25Nico and 0.5Nico groups that exhibited consistent regulatory trends with those observed in young mice. The results highlighted major changes in amino acid metabolism, glycolysis, and fatty acid metabolism, particularly in WAT and muscle, suggesting enhanced energy expenditure that may underlie the improved motor performance seen in nicotine‐treated mice (Figure 4D).

To clarify the relationship between behavioral outcomes and metabolic changes, correlation analyses were performed between significantly altered metabolites and locomotor behaviors across multiple organs in the 0.5Nico group. Behavioral assessments revealed a significant increase in total distance traveled in the OFT (Figure 1C), as well as enhanced rotarod performance at 25 rpm (Figure 1H). Additional improvements were observed in motor‐related parameters, including frame distance, movement speed (back), movement energy (back), and running capacity. Emotional behavior indicators—such as the center time ratio and body length—were also positively influenced by nicotine treatment. Notably, 3D behavioral analysis at 22 months demonstrated that long‐term nicotine intake reversed age‐related motor pattern decline in the 0.5Nico group compared to Saccharin controls. Recovered behaviors included falling with a right turn, rearing, trotting and exploring, walking while sniffing with the head down, walking while sniffing with a left or right turn, and pausing followed by sniffing. A correlation heatmap was also generated to assess associations between behavioral phenotypes and metabolic parameters, including nicotine and cotinine levels across multiple tissues (Figure 4D), further supporting a link between metabolic modulation and improved motor outcomes in aged mice.

In the liver, D‐PA showed a positive correlation with the total distance traveled in the OFT at 22 months, as well as with nicotine and cotinine levels. In contrast, 13‐cis‐retinal exhibited significant negative correlations with multiple locomotor parameters, including total distance (22‐month OFT), frame distance, movement speed (back), movement energy (back), trotting and exploring behavior, walking while sniffing with the head down, and cotinine levels. In skeletal muscle, 3‐MH was significantly negatively correlated with 22‐month OFT total distance, trotting and exploring behavior, and both nicotine and cotinine levels. Tyr also showed strong negative correlations with 22‐month OFT total distance, frame distance, movement speed (back), movement energy (back), running, trotting and exploring behavior, and nicotine/cotinine levels. Additionally, α‐KG was negatively correlated with total distance, rotarod performance (25 rpm), movement speed (back), movement energy (back), running, and trotting and exploring behavior. In the pancreas, PC(22:5) was significantly negatively correlated with 22‐month OFT total distance. In plasma, SM(d18:1/22:0) exhibited positive correlations with 22‐month OFT total distance and frame distance, whereas TEA was negatively correlated with OFT total distance. PGE_2_ showed a negative correlation with walking while sniffing with the head down, and a positive correlation with pausing followed by sniffing. Several metabolites—including PC(22:6), DHCA, TA, and PGE_2_—were negatively correlated with cotinine levels, whereas PC(15:0/16:0) and TA were positively correlated. PC(22:6), TA, and PGE_2_ also exhibited negative correlations with nicotine levels, though TA uniquely showed a positive correlation with nicotine as well. In WAT, both Pro and LysoPC(16:1/0:0) were negatively correlated with multiple motor performance metrics, including 22‐month OFT total distance, frame distance, movement speed (back), movement energy (back), trotting and exploring, and walking while sniffing with the head down. Pro was also negatively associated with rotarod performance at 22 months. Additionally, LysoPC(18:1), IQ, and G6P were negatively correlated with movement speed (back), movement energy (back), and nicotine levels. LysoPC(18:1) further showed negative correlations with running, trotting and exploring, and walking while sniffing with the head down behaviors. Notably, several of these behavior‐associated metabolites are involved in distinct metabolic pathways. In muscle, α‐KG participates in pentose and glucuronate interconversion. In the pancreas, PC(22:5) is involved in glycerophospholipid metabolism and alpha‐linolenic, linoleic, and arachidonic acid pathways. In plasma, SM(d18:1/22:0) is linked to sphingolipid metabolism, while TEA is associated with glycerophospholipid metabolism. In WAT, G6P plays roles in insulin secretion, insulin resistance, inositol phosphate metabolism, starch and sucrose metabolism, and diabetic cardiomyopathy. LysoPC(16:1) is also linked to glycerophospholipid metabolism (Figure 4D). Overall, these findings suggest that nicotine exerts its effects on motor function in aged mice by reshaping metabolic networks, primarily through glucose and lipid‐related pathways. Among the examined organs, WAT emerges as a central mediator of nicotine‐induced metabolic reprogramming during aging, underscored by the abundance and significance of its metabolite associations.

Given that long‐term administration of 0.5 g L^−1^ nicotine (0.5Nico) was associated with increased physical activity in mice, we investigated differential metabolite profiles across various organs in the Young, 0.5Nico, and Old groups, and assessed their relationships with exercise distance, as well as nicotine and cotinine levels. In the liver, levels of D‐PA were significantly elevated in both the Young and 0.5Nico groups but markedly reduced in the Old group, showing a strong positive correlation with both nicotine and cotinine concentrations. In muscle tissue, 3‐MH and Tyr levels were significantly lower in the Young and 0.5Nico groups and elevated in the Old group, with both metabolites negatively correlated with nicotine and cotinine levels. Similarly, in WAT, G6P levels were reduced in the Young and 0.5Nico groups and elevated in the Old group, also exhibiting negative correlations with nicotine and cotinine (Table S6, Supporting Information).

Declines in motor function and metabolic activity are recognized hallmarks of aging.^[^ ^44^ ^]^ To comprehensively assess aging and the effects of long‐term oral nicotine exposure, we developed a composite Z‐score, termed the Behavior‐Metabolome Age Score (BMAge score), to quantify biological aging (Figure 4E,F). This score integrated results from conventional behavioral assays, a hierarchical 3D‐motion capture framework for mapping spontaneous behaviors (Figure 4E), and metabolite profiles across multiple tissues (Figure 4F). Radar plots of Z‐scores for behavioral and metabolomic parameters revealed that the 0.5Nico group exhibited profiles closely resembling those of the Young group, with the 0.25Nico group showing intermediate similarity. In contrast, the Old group demonstrated the greatest divergence from the Young phenotype (Figure 4E,F). Behavioral and metabolomic Z‐scores exhibited consistent directional trends (Figure 4G). Collectively, these findings suggest that long‐term oral nicotine consumption ameliorates aging‐associated phenotypes, primarily through modulation of energy metabolism.

### 2.7 Chronic Oral Nicotine Modulates Gut Microbiota and Metabolomic Dynamics During Aging

**Figure 5:**
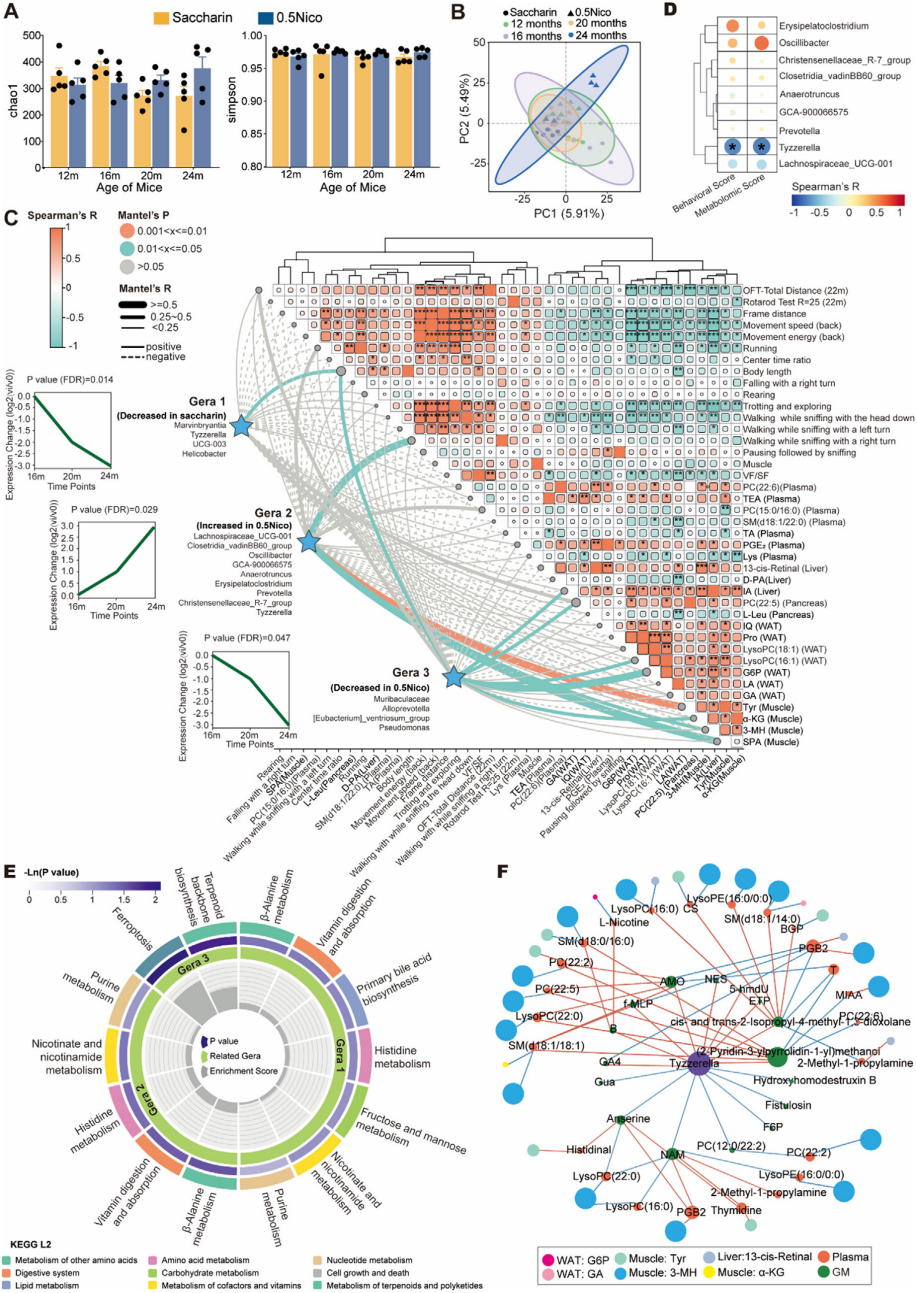
Dynamic changes and metabolomic profiling of gut microbiota and the correlation networks in relation to aging under nicotine administration. A) Alpha diversity in the Saccharin and 0.5Nico groups at the ages of 12, 16, 20, and 24 months old (Left = Chao1, right = Simpson). B) PCA plot of the gut microbiota composition in the Saccharin and 0.5Nico group at 12, 16, 20, and 24 months of age. C) Mantel test‐based correlation heatmap illustrating associations between clustered microbial genera and differential metabolites, locomotor phenotypes, and adipose tissue index in the 0.5 g L^−1^ nicotine‐treated group. The developmental trajectory of gut microbiota is shown as relative genus‐level abundance in Saccharin‐ and 0.5Nico‐treated mice from 16 to 24 months. Gera1 was significantly downregulated in the Saccharin group, while Gera2 exhibited a progressive increase and Gera3 a consistent decline in the 0.5Nico group. D) Spearman correlation heatmap demonstrating the relationship between microbial cluster Gera2 and BMAge scores. E) KEGG pathway enrichment analysis of differential metabolites correlated with clustered microbial genera. F. Correlation network illustrating metabolite associations between the *Tyzzerella* genus and plasma, as well as other organ systems. Multiple Mann–Whitney U tests were used in (A), and STEM (Short Time‐series Expression Miner) analysis was applied in (C). Data was shown as mean ± s.e.m. *p < 0.05, **p < 0.01, ***p < 0.001. PC(22:6), PC(22:6(4Z,7Z,10Z,13Z,16Z,19Z)/18:1(11Z)); TEA, Triethanolamine; TA, Traumatic acid; PGE_2_, Prostaglandin E2; Lys, L‐Lysine; D‐PA, D‐pantothenic acid; IA, Itaconic acid; PC(22:5), PC(22:5(7Z,10Z,13Z,16Z,19Z)/18:1(11Z)); L‐Leu, L‐Leucine; IQ, Indole‐5,6‐quinone; Pro, L−Proline; LysoPC(18:1), LysoPC(18:1(9Z)); LysoPC(16:1), LysoPC (16:1(9Z)/0:0); G6P, Glucose 6‐phosphate; LA, L‐Lactic acid; GA, Glycolic acid; Tyr, L‐Tyrosine;α‐KG, oxoglutaric acid; 3‐MH, 3‐methylhistidine; SPA, Sphinganine; PC(22:2), PC(22:2(13Z,16Z)/16:1(9Z)); CS, Cholesterol sulfate; BGP, beta‐glycerophosphate; PGB_2_, ProstaglandinB2; T, Thymidine; MIAA, methylindole‐3‐acetate; F6P, fructose‐6‐phosphate.

The dynamic alterations in the gut microbiome throughout aging, along with their involvement in age‐related pathologies, have been widely acknowledged.^[^ ^28^, ^45^ ^]^ The composition and community structure of the gut microbiota are now recognized as critical indicators of health and physiological homeostasis.^[^ ^1^, ^28^ ^]^ Since oral nicotine delivery through drinking water directly affects the gastrointestinal tract, this study examined the consequent alterations in the gut microbiota. To investigate the dynamic, multi‐timepoint changes in the gut microbiota of aging mice receiving 0.5 g L^−1^ nicotine in drinking water—especially those exhibiting significant behavioral and metabolomic improvements—we performed Short Time‐series Expression Miner (STEM) analysis. Longitudinal 16S rRNA sequencing of fecal samples was conducted at 12, 16, 20, and 24 months of age to characterize the effects of prolonged nicotine exposure on microbial composition. Alpha diversity analysis indicated no significant changes in overall microbial richness or diversity following nicotine treatment (**Figure** **5**A; Figure S9A, Supporting Information). However, principal coordinate analysis (PCoA) based on species abundance revealed distinct clustering of microbial communities, especially in 24‐month‐old mice, suggesting age‐associated shifts modulated by nicotine exposure (Figure 5B). At the genus level, compositional profiling of the top 30 taxa (Figure S9B, Supporting Information) showed marked differences between the Saccharin and 0.5Nico groups, particularly in the relative abundance of *Muribaculaceae*, *Lachnospiraceae_NK4A136_group*, *Alistipes*, *Lactobacillus*, and *Bifidobacterium*, indicating nicotine‐induced alterations in gut microbial community structure.

STEM analysis was conducted between 16 and 24 months of age, encompassing the critical period ≈ 18 months when key behavioral changes were observed, to identify age‐related shifts in the gut microbiota. A significance threshold of P < 0.05 was applied to detect dynamic trends, and bacterial taxa exhibiting these patterns were classified accordingly. Three significant temporal patterns emerged: Gera1, characterized by a consistent decrease in the Saccharin group; Gera2, showing a steady increase in the 0.5Nico group; and Gera3, demonstrating a consistent decline in the 0.5Nico group. Notably, both Gera2 and Gera3 were significantly correlated with expression levels of amino acids, lipids, and glucose in WAT and muscle, underscoring a metabolic linkage between these microbial shifts (Figure 5C).

Nicotine‐upregulated genera were primarily classified within the *Firmicutes* and *Proteobacteria* phyla, whereas nicotine‐downregulated genera were mainly assigned to the *Bacteroidetes* phylum (Figure S9C, Supporting Information). The *Firmicutes*‐to‐*Bacteroidetes* (Fir/Bac) ratio, previously reported as a potential indicator of gut microbial homeostasis relevant to obesity, metabolism, and aging,^[^ ^46^, ^47^, ^48^ ^]^ showed a significant difference between nicotine and saccharin groups at 16 months. Importantly, nicotine‐treated mice maintained a higher Fir/Bac ratio compared to controls throughout aging, indicating that long‐term high‐dose nicotine intake modulates dynamic gut microbial homeostasis, as demonstrated by phylum‐level shifts detectable from 16 months onward (Figure S9D,E, Supporting Information). These findings align with recent reports describing nonlinear microbial dynamics during aging.^[^ ^49^ ^]^ Notably, *Tyzzerella* was identified as the genus overlapping between Gera1 and Gera2 patterns and exhibited a significant negative correlation with the BMAge score, supporting nicotine's anti‐aging effects (P _Behavioral Score_ = 0.019, P _Metabolomic Score_ = 0.024, Spearman's correlation; Figure 5D). Collectively, these results suggest that the anti‐aging benefits of lifelong 0.5 g L^−1^ nicotine administration on behavioral and metabolomic phenotypes are associated with the preservation of *Tyzzerella* abundance and the maintenance of gut microbial homeostasis.

To investigate gut microbiota‐derived metabolic functions in aged mice following nicotine administration, untargeted metabolomic profiling of fecal samples was performed using LC‐MS. Lifelong oral nicotine exposure induced substantial alterations in the gut metabolome, with 147 metabolites significantly upregulated and 157 downregulated in the 0.5Nico group compared to saccharin controls (Figure S10A,B, Supporting Information). These differential metabolites indicated notable shifts in energy metabolism. Specifically, levels of nicotinamide (NAM), glucose–pyruvate–lactate, and anserine were decreased, whereas cotinine, indole acetaldehyde, tyrosinamide, and the tripeptide Ala‐Leu‐Trp were elevated in the 0.5Nico group (Figure S10C, Supporting Information). Pathway enrichment analysis revealed significant involvement of amino acid metabolism, nicotinate and nicotinamide metabolism, sphingolipid signaling, and choline‐related pathways (Figure S10D, Supporting Information), underscoring extensive reprogramming of microbial metabolic activity in response to long‐term nicotine intake.

Moreover, nicotine‐associated microbial clusters Gera1 and Gera2 exhibited significant correlations with differential metabolites enriched in nutrient absorption pathways, whereas Gera3 was linked to ferroptosis‐related pathways, implying a potential role in apoptosis regulation (Figure 5E). Additionally, microbial metabolites correlated with the *Tyzzerella* genus showed strong associations with elevated plasma sphingomyelin levels and key organ‐specific biomarkers (identified metabolites with AUC > 0.85, Figure 3) (Figure 5F). Collectively, these findings suggest that nicotine‐modulated gut microbiota in aged mice may influence systemic metabolic homeostasis, potentially via sphingolipid signaling pathways.

### 2.8 Nicotine Modulates the Sphingolipid Metabolic Pathway

**Figure 6:**
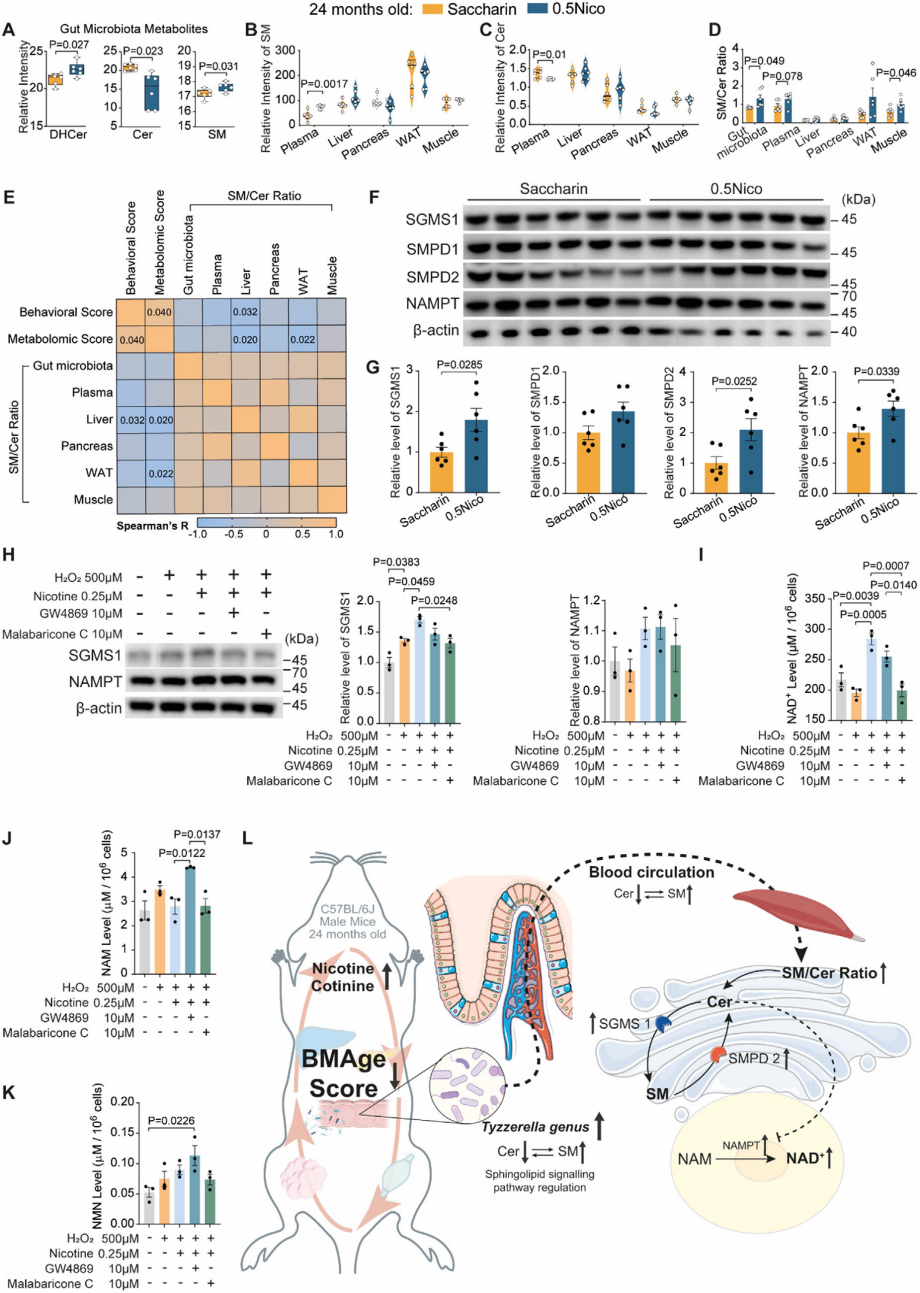
Regulation of sphingolipid metabolism and associated pathways in aged mice following oral nicotine administration. A) Expression levels of sphingolipid metabolites derived from gut microbiota in aged mice. B,C) Quantification of sphingomyelin (B) and ceramide (C) levels in plasma, liver, pancreas, WAT, and muscle of aged mice. D) SM/Cer ratio measured in gut microbiota, plasma, liver, pancreas, WAT, and muscle from Saccharin and 0.5Nico groups. E) Spearman correlation heatmap depicting associations between BMAge scores and SM/Cer ratios across different tissues in Saccharin and 0.5Nico groups. F) Western blot analysis of SGMS1, SMPD1, SMPD2, and NAMPT in muscle tissue from Saccharin and 0.5Nico groups; β‐actin served as the loading control. G) Quantification of relative protein expression levels of SGMS1, SMPD1, SMPD2, and NAMPT in muscle tissue from Saccharin and 0.5Nico groups. H) Western blot analysis of SGMS1 and NAMPT in C2C12 cells following H_2_O_2_‐induced oxidative stress, with or without nicotine and inhibitors of SGMS and nSMase pretreatment; β‐actin used as loading control. I–K) HPLC quantification of NAD⁺ (I), NAM (J), and NMN (K) levels in C2C12 cells exposed to H_2_O_2_‐induced oxidative stress with or without nicotine and SGMS/nSMase inhibitor pretreatment. L) Schematic overview of sphingolipid metabolic regulation following long‐term oral nicotine administration in aged mice. Welch's t‐test was applied in (A); two‐tailed multiple t‐tests with FDR correction in (B–D); Student's *t*‐test in (G); one‐way ANOVA with Tukey's multiple comparisons in (H–K). Data was shown as the mean ± s.e.m., n = 3‐6 in each group. SM, sphingomyelin; Cer, ceramide; SGMS, sphingomyelin synthase; SMPD, sphingomyelin phosphodiesterase; nSMase, neutral sphingomyelinases; NAMPT, nicotinamide phosphoribosyl transferase; NAD^+^, nicotinamide adenine dinucleotide; NAM, nicotinamide; NMN, β‐nicotinamide mononucleotide; BMAge, Behavior‐Metabolome Age scoring; HPLC, high performance liquid chromatography.

To elucidate the role of gut microbiota in nicotine‐induced metabolic regulation during aging—with a focus on sphingolipid metabolism—we analyzed metabolites involved in this pathway. Strikingly, gut microbiota analysis revealed that 0.5Nico treatment significant altered the sphingolipid pathway, with a decrease in ceramide (Cer) and increase in sphingomyelin (SM) and dihydroceramide (DHCer) levels (P _DHCer_ = 0.027, P _Cer_ = 0.023, P _SM_ = 0.031, 0.5Nico vs Saccharin, two‐tailed unpaired *t*‐test; **Figure** **6**A). Cer has recently been identified as a hallmark of aging and a critical mediator of age‐associated metabolic disorders.^[^ ^11^ ^]^ Notably, these alterations were consistently observed across multiple tissues. Plasma samples from 0.5Nico‐treated mice also exhibited markedly reduced Cer levels along with increased SM levels (P _SM_ = 0.0017, P _Cer_ = 0.01, 0.5Nico vs Saccharin, two‐tailed multiple unpaired *t*‐test with false discovery rate (FDR) correction; Figure 6B,C). The SM/Cer ratio was consistently elevated across gut microbiota, plasma, and muscle in 0.5Nico‐treated aged mice (P _Gut microbiota_ = 0.049, P _Plasma_ = 0.078, P _Muscle_ = 0.046, 0.5Nico vs Saccharin, two‐tailed multiple unpaired *t*‐test with FDR correction; Figure 6D). Importantly, this ratio demonstrated a significant negative correlation with the BMAge score (Figure 6E), suggesting its potential as an aging‐related biomarker.

To delineate the molecular basis of nicotine's effects on sphingolipid metabolism during aging, we examined protein expression of key regulatory enzymes in muscle tissue via western blot, including sphingomyelin synthases (SGMS), acid sphingomyelinase (aSMase), and neutral sphingomyelinases (nSMase).^[^ ^50^ ^]^ Nicotine administration significantly upregulated the expression of SGMS1 and sphingomyelin phosphodiesterase 2 (SMPD2, encoding nSMase), but did not affect SMPD1 (encoding aSMase), indicating that oral nicotine intake induces substantial remodeling of sphingolipid metabolism. Meanwhile, expression of nicotinamide phosphoribosyltransferase (NAMPT)—a key enzyme in NAD⁺ biosynthesis^[^ ^10^ ^]^ was also significantly increased (P _SGMS1_ = 0.0285, P _SMPD2_ = 0.0252, P _NAMPT_ = 0.0339, 0.5Nico vs Saccharin, two‐tailed unpaired *t*‐test; Figure 6F,G), aligning with our previous findings of elevated NAD⁺ levels in the muscle of 0.5Nico‐treated mice (Figure 4B). These results suggest a coordinated regulation of sphingolipid and NAD⁺ metabolism.

To investigate the functional roles of SGMS and nSMase in nicotine‐mediated metabolic regulation during aging, we established an in vitro model using C2C12 myoblasts subjected to H_2_O_2_‐induced oxidative stress. Cells were pretreated with nicotine (0.2–1 µM)^[^ ^51^, ^52^, ^53^ ^]^ under oxidative stress, along with pharmacological modulation of enzyme activity (Figure S11A, Supporting Information). Based on our in vivo findings—demonstrating nicotine‐mediated protection against age‐related motor decline and associated changes in sphingolipid and NAD⁺ metabolism—0.25 µM nicotine pretreatment, which effectively recapitulated key metabolic features observed in aged mice, was selected for downstream pharmacological assays (Figure S11A, Supporting Information). Two inhibitors, Malabaricone C (SGMS inhibitor)^[^ ^54^ ^]^ and GW4869 (nSMase inhibitor),^[^ ^33^, ^55^ ^]^ were applied to examine their roles in modulating nicotine's effects under oxidative stress. Malabaricone C efficiently suppressed SGMS1 expression, blocking the conversion of SM to Cer (P = 0.0248, Figure 6H; Figure S11B–D, Supporting Information), identifying SGMS as a critical regulatory node in nicotine‐mediated sphingolipid remodeling. Moreover, inhibition of SGMS in nicotine‐pretreated cells significantly reduced NAD⁺ levels, whereas inhibition of nSMase did not produce a similar effect—likely due to a ceiling effect in nicotine‐induced NAD⁺ elevation, beyond which further enhancement was not achievable (P = 0.0007, Figure 6I). Interestingly, nSMase inhibition led to increased levels of NMN and NAM (Figure 6J,K), suggesting that, in addition to its role in regulating SM‐to‐Cer conversion, nSMase may contribute to NAD⁺ biosynthesis by modulating precursor availability. Neither inhibitor had a significant impact on mitochondrial superoxide production following oxidative stress in nicotine‐pretreated cells (Figure S11E, Supporting Information). Collectively, our in vivo and in vitro findings demonstrate that nicotine enhances NAD⁺ availability and promotes energy metabolism in aged mice by modulating sphingolipid turnover through regulation of SGMS and nSMase (Figure 6L). Given the critical role of sphingolipid pathways in sustaining NAD⁺ homeostasis, potentially through Sirtuin 1 (SIRT1),^[^ ^56^ ^]^ long‐term oral nicotine administration reduced SM‐to‐Cer conversion and limited Cer accumulation, particularly in plasma and muscle. This metabolic reprogramming (Figure S11F, Supporting Information) correlated with improved motor performance, decreased BMAge score, elevated NAD⁺ levels, and enhanced energy utilization, which mirrored phenotypes observed in young mice (Figure 6L).

## 3 Discussion

This study demonstrates that lifelong oral nicotine administration attenuates age‐related motor decline in mice without inducing detectable toxicity in major metabolic organs or impairing immune function. Integrated behavioral and metabolomic analyses reveal that nicotine reprograms systemic energy and glycolipid metabolism, preserves gut microbial homeostasis, and modulates the sphingolipid pathway—characterized by reduced ceramide and increased sphingomyelin levels—thereby enhancing NAD^+^ availability and metabolic resilience. These effects collectively contribute to a biologically younger phenotype, as reflected by the BMAge score. Distinct from inhaled forms, oral nicotine may thus serve as a modulatory agent in aging‐associated metabolic remodeling.

Numerous studies have reported conflicting effects of nicotine on aging‐related phenotypes, often determined by the route, dosage, and duration of exposure. For example, transdermal nicotine at 15 mg per day for 6 months improved attention and memory in nonsmoking patients with amnestic mild cognitive impairment,^[^ ^57^ ^]^ while intraperitoneal injection of 0.04 mg/kg for 7 days enhanced learning in aged SAM‐P/8 mice.^[^ ^58^ ^]^ In contrast, 100–200 µg mL^−1^ delivered via drinking water for 28 days have been shown to induce β‐cell senescence and impair glucose metabolism in diabetic mice.^[^ ^59^ ^]^ Similarly, repeated intraperitoneal injection of 2 mg kg^−1^ per day (administered four times daily) for 5 weeks led to spermatogonia stem cell senescence in mice.^[^ ^60^ ^]^ These findings emphasize that nicotine's biological actions are highly context‐dependent.

Given nicotine's context‐dependent effects, we employed a long‐term oral administration paradigm spanning 22 months, simulating voluntary intake and avoiding stress associated with injections or pump implantation. Saccharin was added at a minimal concentration (0.2%) to mask nicotine's bitter taste and maintain fluid intake; both our behavioral and metabolomic analyses confirmed that saccharin alone exerted no measurable impact. Pharmacokinetic evidence supports the relevance and safety of our dosing strategy. The nicotine concentrations used (0.25 and 0.5 g L^−1^) yield plasma cotinine levels comparable to those observed in moderate to heavy human smokers.^[^ ^61^, ^62^ ^]^ These doses fall below the oral LD_50_ threshold in mice and are aligned with previous long‐term exposure studies.^[^ ^63^ ^]^ It is worth noting that oral administration produces distinct pharmacokinetics compared to inhaled nicotine, likely contributing to the differential physiological outcomes observed in our model.

Our data show that oral nicotine improves locomotor endurance and spontaneous activity in aging mice in a dose‐dependent manner. This is consistent with prior literature indicating that aging is accompanied by reduced physical activity^[^ ^64^ ^]^ and mitochondrial decline,^[^ ^65^ ^]^ and that metabolic treatments can mitigate these effects.^[^ ^66^ ^]^ Notably, nicotine‐induced improvements in motor function were paralleled by enhanced energy metabolism in skeletal muscle and WAT—two organs critically involved in aging‐related metabolic regulation. WAT emerged as a central node in nicotine‐responsive metabolic networks, accompanied by increased substrate utilization, elevated NAD^+^ and NMN levels in the muscle, which have strong correlations with improved behavioral outcomes and no signs of mitochondrial dysfunction, supporting nicotine's role in promoting peripheral metabolic resilience.

In contrast to motor effects, nicotine did not significantly alter cognitive performance in NOR and Y‐maze tests. This aligns with previous studies showing that nicotine's cognitive effects are highly variable, depending on dose, treatment duration, and cognitive baseline.^[^ ^58^, ^67^, ^68^, ^69^ ^]^ While acute or low‐dose nicotine may improve attention and memory in some models,^[^ ^70^, ^71^, ^72^, ^73^ ^]^ chronic exposure often leads to tolerance.^[^ ^57^, ^74^, ^75^, ^76^ ^]^ Our findings align with prior reports that nicotine's benefits are most pronounced in impaired systems,^[^ ^67^, ^77^, ^78^ ^]^ as evidenced by its selective enhancement of motor function in aged mice without altering cognition in healthy aged subjects. It is important to note that nicotine's effects are highly age‐dependent; while potentially neuroprotective in aging, nicotine exposure during neurodevelopment can be neurotoxic.^[^ ^79^ ^]^

We focused our toxicological assessments on the liver, pancreas, WAT, and muscle due to their central roles in energy regulation and aging.^[^ ^80^ ^]^ Prior studies have shown that nicotine alters lipid metabolism in the liver,^[^ ^33^ ^]^ impairs β‐cell function in the pancreas,^[^ ^39^, ^59^ ^]^ disrupts insulin signaling in muscle, and modulates lipolysis in adipose tissue.^[^ ^81^, ^82^ ^]^ Our findings suggest that systemic metabolic alterations induced by long‐term oral nicotine administration may compensate for organ toxicity, thereby contributing to the observed functional benefits.

Time‐resolved gut microbiota profiling uncovered a non‐linear shift in microbial composition ≈ 16 months of age, indicating a critical window for aging‐associated microbial remodeling. Oral nicotine preserved microbiota stability across this transition, particularly maintaining the *Fir/Bac* ratio, a recognized aging and diseases biomarker^[^ ^46^, ^47^, ^48^ ^]^; and enriched *Tyzzerella*, which correlated with improved muscle/WAT metabolism and reduced BMAge scores. Metabolomic profiling further identified increased levels of microbial‐derived metabolites (e.g., cotinine, indole acetaldehyde, and Ala‐Leu‐Trp), linked to nicotinate, amino acid, and sphingolipid pathways. Mechanistically, these changes converge on a gut microbiota–sphingolipid–NAD⁺ axis: nicotine elevated the SM/Cer ratio in the gut, plasma, and muscle, reduced age‐related ceramide accumulation, and upregulated SGMS1 and SMPD2, enhancing NAD⁺ biosynthesis and energy metabolism. In vitro inhibition of SMS and nSMase abolished nicotine‐induced NAD⁺ elevation, supporting sphingolipid turnover as a key metabolic regulator during aging.

We acknowledge that our study also has several limitations. First, our study used only male mice; potential sex‐specific responses remain to be addressed. Second, although BMAge offers a novel integrative index of biological aging, its broader applicability requires validation across independent cohorts. Third, although oral nicotine did not induce toxicity in our setting, its known addictive^[^ ^83^ ^]^ and cardiovascular risks^[^ ^84^ ^]^ in humans cannot be overlooked. Nicotine activates mesolimbic dopaminergic circuits, raising concerns regarding dependence and adverse metabolic outcomes. Moreover, oral administration differs significantly from human smoking or vaping in pharmacokinetics and addictive potential; thus, caution is required when extrapolating these findings.

In summary, our findings reveal a previously underappreciated role for oral nicotine in promoting systemic metabolic resilience and preserving motor function during aging. Through a coordinated reprogramming of peripheral metabolism and gut microbiota—centered on sphingolipid and NAD⁺ regulation—nicotine confers systemic resilience against age‐related decline. While nicotine's risks must be carefully weighed, our study suggests that under controlled conditions, long‐term oral nicotine may modulate aging‐related phenotypes through mechanisms distinct from its addictive properties. Future work should aim to dissect these molecular pathways and assess alternative non‐addictive cholinergic agents that recapitulate nicotine's beneficial effects on aging. In particular, given the involvement of midbrain dopaminergic circuits in motor regulation and the emerging evidence that the microbiota–gut–brain axis modulates physical activity via dopaminergic signaling,^[^ ^85^ ^]^ future studies is required to delineate the central and peripheral mechanisms of nicotine action and investigate how peripheral metabolic cues may influence neurocircuitry associated with aging‐related functional decline.

## 4 Experimental Section

### Animals

C57BL/6J male SPF mice (7 weeks of age) were purchased from Beijing Vital River Laboratory Animal Technology Co., Ltd. and used for nicotine administration. Mice were group‐housed five per cage under a 12/12 h light/dark cycle with free access to food and water, maintained at 22–25 °C room temperature and 50–60% humidity. Water and food consumption were measured weekly per cage, while body weights were measured of each individual mouse. All experiments were conducted during the light phase of the cycle. In this study, 24‐month‐old mice were used as the natural aging model, with 8‐month‐old mice serving as the young controls. For euthanasia, 24‐month‐old mice were anesthetized with an intraperitoneal injection of 1% pentobarbital sodium (in 0.9% saline) administered at a dose of 100 mg kg^−1^. All animal studies and experimental procedures were approved by the Animal Care and Use Committee at the Shenzhen Institute of Advanced Technology, Chinese Academy of Sciences, in full compliance with the ARRIVE guidelines (SIAT‐IACUC‐220914‐NS‐CZX‐A2187).

### Cell Culture

In this study, the C2C12 mouse myoblast cell line supplied by the Shanghai Cell Bank of the Chinese Academy of Sciences (Shanghai, China). Based on a previous study. C2C12 cells were cultured in Dulbecco's modified Eagle's growth medium containing 10% heat‐inactivated fetal bovine serum (FBS) and 1% penicillin/streptomycin, and maintained in a humidified atmosphere with 5% carbon dioxide (CO_2_) at 37 °C.

### Nicotine Administration in Animals

Oral nicotine administration via water supply began at eight weeks of age. In this study, sodium saccharin was used as a vehicle control, while water was used as a complete blank control. In the nicotine treatment groups, nicotine ditartrate (Sigma‐Aldrich, CAS#65‐31‐6) was dissolved in sterilized water containing 0.2% saccharin, which was commonly used to mask the bitter taste of nicotine. The final concentration of nicotine was 0.25 g L^−1^ for the low‐dose group, and 0.5 g L^−1^ for the high‐dose group (0.5Nico). The pH of the solutions was adjusted to 7.0–7.1. This design allowed investigation of the lifelong effects of nicotine on aging while controlling for the potential impact of sodium saccharin.^[^ ^86^ ^]^

### Nicotine for Cell Cultures Study

Nicotine was first dissolved in sterile double‐distilled water (ddH_2_O) to prepare a 10 mm stock solution. The solution was then filter‐sterilized and diluted in the growth medium to final working concentrations of 0.25, 0.5, and 1 µm. Vehicle control cells received an equivalent volume of sterile ddH_2_O. A 2‐h nicotine pre‐treatment was performed prior to oxidative stress induction, under standard cell culture conditions (37 °C, 5% CO_2_, humidified atmosphere).

### Oxidative Stress Model in C2C12 Cells

Oxidative stress was induced using hydrogen peroxide (H_2_O_2_) at a final concentration of 500 µmol L^−1^, as previously described.^[^ ^87^ ^]^ A working solution of H_2_O_2_ was prepared by diluting filtered 30% H_2_O_2_ with sterile PBS at a ratio of 1:199, followed by further dilution in growth medium to achieve the final concentration. The control group received PBS alone. Following nicotine pre‐treatment, C2C12 cells were exposed to the H_2_O_2_‐containing medium for 1 h under standard culture conditions (37 °C, 5% CO_2_, humidified atmosphere).

### Drug Treatment

GW4869 (MCE, CAS#6823‐69‐4) and Malabaricone C (MCE, CAS#63335‐25‐1) were used in cell‐based experiments. Both compounds were initially dissolved in DMSO at a stock concentration of 10 mm with the aid of ultrasonic treatment. They were subsequently diluted in nicotine‐containing growth medium to a final working concentration of 10 µm, as previously reported.^[^ ^54^, ^55^ ^]^ The vehicle control group received an equivalent volume of DMSO.

### Fecal Sample Collection

Feces were collected from mice at 12, 16, 20, and 24 months of age, with sterilized forceps and 1.5 mL Eppendorf tube, and were immediately snap frozen in liquid nitrogen, then stored at ‐80 °C. Samples for 16S rRNA sequencing and analysis were directly sent to OE Biotech. Co., Ltd., (Shanghai, China).

### Blood Collection

During terminal procedure, mouse eyeballs were swiftly removed with fine forceps following anesthesia. Whole blood was collected into EP tubes containing EDTA, then kept on fresh ice. After 30 mins, blood plasmas were centrifuged at 4 °C at 10,000 rpm, and the resulting plasma was stored at ‐80 °C until analysis.

### Fresh Tissue Dissection and Tissue Weighing

Mice were sacrificed at 24 months of age, and fresh tissues were harvested post‐anesthesia. Tissues were placed in 1.5 mL tubes and snap‐frozen in liquid nitrogen, and stored at ‐80 °C until analysis. Liver, pancreas, skeletal muscles, WAT (including SF, PF, and EF), and BAT were collected. Gastrocnemius muscles were weighed bilaterally, wherwas WAT and BAT were weighed unilaterally.

### Behavioral Tests

The open field test, novel object recognition test, and Y‐maze test were conducted at 14, 18, and 22 months of age. The pole test, wire suspension test, and constant‐rotarod test were performed at 18 and 22 months. 3D spontaneous behavior tracking was conducted at 24 months of age before sacrifice.

### OFT

The OFT was conducted in a noise‐reduced animal behavior room. The open field apparatus was 50 cm × 50 cm × 50 cm (length × width × height). A video camera was mounted directly above the apparatus to record mouse trajectories. At the start of the test, each mouse was gently placed at the center of the arena and allowed to explore freely for 10 min. After the test, the mouse was returned to its home cage. Video recordings were analyzed using VisuTrack software (Shanghai Xinruan Information Technology Co., Ltd.). The last 5 min of each session were used for analysis. The following parameters were measured: total distance traveled, average velocity, number of entries into the central area, time spent in the center, and the ratio of center distance to total distance. The ratio of center movement to total movement was statistically analyzed.

*NOR*

The NOR test was used to examine cognitive memory in aging mice. 24 hours after the mice finished the OFT, stick two identical objects with the diameter of 2 cm in the midpoint of two of the four quadrants of the open field box on the same side of the box, and let the mice explore freely for 10 minutes before putting them back. After raising the cage, 75% alcohol is used to remove the smell of the box and objects; replace one of the objects after an interval of 2 hours, and then put the same mouse in it and let it explore. During both training and testing, the camera directly above the box was used to record the mouse's movement trajectory, and then computer behavior software was used to analyze the mouse's head trajectory within 10 minutes during the testing period. Calculate the exploration index (Discrimination index, DI) of each animal based on the mouse's total object exploration time (Tt), time to explore new objects (Tn), and time to explore old objects (To). The formula is as follows:

(1) $$\mathrm{DI}=\frac{\mathrm{Tn}-\mathrm{To}}{\mathrm{Tt}}$$

### Y‐Maze Test

The Y‐maze test was used to assess spatial working memory in aged mice through spontaneous alternation behavior (SAB). The apparatus consisted of three opaque arms (labeled A, B, and C), arranged at 120° angles, each measuring 30 cm in length, 7 cm in width, and 15 cm in height. The maze was placed in an SPF‐level barrier facility with soft lighting, white noise background, and fixed spatial cues around the room. A camera was positioned directly above the maze to record movement trajectories. Before each test, the maze was cleaned with 75% ethanol and allowed to dry. Each mouse was placed at the center of the maze facing Arm A and allowed to explore freely for 8 min. An arm entry was defined as all four limbs entering at least one‐third of the arm's length. A correct alternation was recorded when the mouse entered three different arms consecutively. For each mouse, the number of correct alternations (n) and total number of arm entries (N) were recorded. The SAB percentage was calculated using the following formula:

(2) $$\begin{array}{c} \mathrm{SAB}=\frac{n}{N-2}\;\times 100\% \end{array}$$

### Pole Test

The pole test was used to evaluate motor coordination and balance. Mice were placed head‐up at the top of a vertical pole (50 cm in height and 1.5 cm in diameter) with a rough surface. The pole was fixed to a stable base (15 cm in diameter). The time taken for the mouse to turn completely downward and descend to the bottom with both forelimbs touching the ground was recorded. Each mouse underwent three trials per day, and the shortest time was used for analysis. Mice were habituated to the apparatus for 3 consecutive days before testing on the 4th day.

### Wire Suspension

The wire suspension test was performed to assess forelimb strength and grip endurance. A horizontal wire was suspended 50 cm above a soft bedding surface to prevent injury. The wire was taut and stable, allowing the mouse to grip with its forelimbs. During the test, each mouse was allowed to hang by its forepaws, and the latency to fall was recorded. Each mouse was tested three times in a day, and the average time was used for analysis. As with other tests, a 3‐day adaptation period was followed by 1 day of testing.

### Rotarod Test

The rotarod test was used to evaluate motor coordination and endurance using the XR‐YLS‐4C rotarod system (Shanghai Xinruan Information Technology Co., Ltd.). Mice were placed on a rotating rod (3 cm in diameter), with five mice tested simultaneously. The testing protocol included 3 days of adaptation and 1 day of formal testing. For 18‐month‐old mice, six speeds (10, 15, 20, 25, 30, and 40 rpm) were tested. Each speed lasted for 10 min per day. For 22‐month‐old mice, three speeds (15, 20, and 25 rpm) were tested for 5 min per speed per day. During adaptation, each mouse experienced each speed once daily. During the test session, each speed was tested once. Mice were returned to the rotarod after each fall, but were withdrawn from that specific speed test after three falls. Latency to the first fall was recorded for each speed on the testing day.

### 3D Behavioral Data Processing

Each mouse was allowed to freely move in a transparent circular open field (50 cm diameter). Four cameras (Intel RealSense D435) were positioned on each of the four sides of the apparatus to synchronously record 30 min of behavior. Following multi‐perspective video recording of mouse behavior, data were processed using the BehaviorAtlas Analyser (BeA) software, following standardized and previously validated protocols.^[^ ^38^ ^]^ The software facilitated the extraction of 3D mouse skeleton by identifying 16 key body parts on each mouse. A comprehensive set of 39 kinematic parameters was calculated (as detailed in Figure S3; Table S1, Supporting Information), enabling precise comparison of movement dynamics under different conditions. Additionally, the frequency of each behavior type (movement fractions was quantified, as detailed in Figure S4; Table S2, Supporting Information) to compare behavior at the sequence level. During the BeA analysis, 2D skeletal trajectories were obtained from each camera view using DeeplabCut‐trained models to track mouse body parts. The distribution and intensity of movements (MI) are visualized using heat maps overlaid on the skeletons, with MI scaled from 0 to 1 and presented in arbitrary units (a.u.). Subsequently, behavior decomposition and unsupervised clustering were performed on the 3D skeleton time‐series data to extract behavioral movement clusters. The behavioral repertoire included locomotor information, represented by velocity and non‐locomotor (NM) movements using limbs or organs without trunk movement, such as grooming, and dynamic time alignment kernel, used to measure the similarity between non‐locomotor movement segments. High‐dimensional NM features were embedded into the 2D NM space using UMAP, which formed the 3D behavioral feature space together with the velocity parameter. Based on the 3D behavior features, unsupervised clustering was applied to cluster behavior types. Behavioral movements were further detected by supervised classification, and the dimensionality reduction data were visualized to visually compare movement patterns between different groups and between different age groups. The 11 behavioral movement fractions or 116 types of transition numbers were embedded into a 2D space using a t‐distributed stochastic neighbor embedding manifold to visualize the high‐dimensional behavioral movement fractions and transitions. Based on this 2D space, a Gaussian kernel function identified the SVM decision boundary to classify groups.

### GTT and ITT

Mice were fasted for 16 h prior to the GTT and 4 h prior to the ITT. For the GTT, mice were injected intraperitoneally with 1.5 g per kg of glucose. For the ITT, mice were injected intraperitoneally with 1.5 UI/kg of insulin. For all tests, blood glucose levels were measured from the tail vein at 0‐, 15‐, 30‐, 60‐, 90‐, and 120‐min post‐injection. GTT and ITT were conducted at 20 months of age.

### Histopathological Examination with Hematoxylin and Eosin (H&E) Staining and Imaging

Tissues fixed in 4% paraformaldehyde were embedded in paraffin and sectioned. Paraffin sections were dewaxed through a graded xylene and ethanol series: xylene I (20 min), xylene II (20 min), absolute ethanol I (5 min), absolute ethanol II (5 min), and 75% ethanol (5 min), followed by washing in distilled water. H&E staining was then performed. Sections were stained with hematoxylin for 3–5 min, rinsed with running water, and differentiated with 1% hydrochloric acid solution. After another rinse, the sections were blued in 0.6–0.7% ammonia water, followed by a brief water rinse. Eosin staining was carried out by immersing the sections in 85% and 95% ethanol for dehydration, followed by eosin staining for 5 min. Final dehydration and clearing steps were performed by sequential incubation in absolute ethanol I (5 min), ethanol II (5 min), ethanol III (5 min), n‐butanol (5 min), xylene I (5 min), and xylene II (5 min). Slides were then air‐dried briefly and mounted with neutral resin. Histological evaluation was performed using a Leica DM2000 LED microscope (Leica Microsystems, Germany).

### Flow Cytometry Staining

Spleens and lymph nodes were harvested and passed through a 70 µm mesh to obtain single‐cell suspensions. Lymphocytes were washed and resuspended in PBS containing 1% FBS. Surface staining was performed in round‐bottom 96‐well plates by incubating cells with fluorophore‐conjugated antibodies for 15 min at room temperature. The antibodies used were: Anti‐mouse CD3‐FITC (Invitrogen); Anti‐mouse CD4‐APC/Cy7 (Invitrogen); Anti‐mouse CD25‐PE (Invitrogen); Anti‐mouse CD8‐PE (Invitrogen); Anti‐mouse NK1.1‐APC (BioLegend); Anti‐mouse CD19‐PerCP/Cy5.5 (BioLegend); Anti‐mouse CD11b‐PE (BioLegend); Anti‐mouse F4/80‐APC (BioLegend); Anti‐mouse Gr‐1‐PE/Cy7 (BioLegend). For FOXP3 intracellular staining, cells were fixed and permeabilized using the FOXP3 Staining Kit (eBioscience) and incubated with anti‐mouse FOXP3‐APC (Invitrogen) for 45 min at 4 °C. For intracellular cytokine staining, lymphocytes were first stimulated with Cell Stimulation Cocktail plus transport inhibitor (Invitrogen) for 5 h. After surface marker staining, cells were fixed and permeabilized using the BioLegend Fixation/Permeabilization Buffer Set, followed by intracellular staining with the following antibodies: IFN‐γ‐PE (BioLegend); IL‐17‐APC (BioLegend); TNF‐α‐PerCP/Cy5.5 (BioLegend). Flow cytometry analysis was performed to quantify the labeled immune cell populations.

### Fecal DNA Extraction and 16S rRNA Sequencing Analysis

Mouse feces were collected and immediately stored at ‐80 °C. For DNA extraction, samples were picked and extracted using a QIAamp PowerFecal DNA Kit (MO BIO Laboratories, Qiagen N.V., Cat No./ID:12830‐50). DNA quality and quantification were assessed with a Biophotometer (Shanghai METASH Instruments, Co. Ltd., B‐500). Bacterial DNA was amplified with the primers targeting V3‐V4 regions (5′‐TACGGRAGGCAG‐3′, 5′‐GGGTATCTAATCCT‐3′). Then, the DNA samples were sent to OE Biotech. Co., Ltd (Shanghai, China) for 16S sequencing. Alignment and demultiplexing of raw 16S ribosomal RNA sequencing data was performed with QIIME 2 2019.7. Primers of the Raw sequence data were cut with Cutadapt via q2‐cutadapt, followed by denoising, and chimera was merged and removed with DADA2 (via q2‐dada2). All amplicon sequence variants (ASVs) from DADA2 were used to construct a phylogeny with fasttree2 (via q2‐phylogeny). And the ASVs were assigned to taxonomy with naïve Bayes classifier (via q2‐feature‐classifier) against the silva database, and an ASV table was created. After normalizing based on the sample with the least sequences, ASVs in each sample with a relative abundance smaller than 0.1% were filtered out. Then, analysis of similarities between different time points of the same mouse was performed using weighted UniFrac or UniFrac distance metric with R phyloseq package.

### STEM Analysis

STEM analysis was used to identify temporal abundance trends in microbial communities. Four trend patterns were examined: continuous increase, continuous decrease, increase‐then‐decrease, and decrease‐then‐increase. Abundance data of bacterial taxa were transformed according to these trend templates. The similarity between the transformed data and each trend pattern was calculated to determine the best‐matching trend for each taxon. To assess statistical significance, time points were randomly permuted and trend analyses were repeated. The expected distribution of bacterial groups in each trend was derived from these permutations, and p‐values were calculated using the hypergeometric distribution.

### Metabolomics Data Preprocessing and Statistical Analysis

Raw LC‐MS data were processed using Progenesis QI software (Waters Corporation, Milford, USA) with the following parameters: precursor tolerance of 5 ppm, fragment tolerance of 10 ppm, and retention time (RT) tolerance of 0.02 min. Internal standard parameters were applied for RT alignment. Isotopic peaks were excluded, noise elimination was set to 10.00, and the minimum intensity threshold was 15% of the base peak intensity. The resulting dataset included 3D data comprising m/z, RT, and peak intensity values. RT–m/z pairs were used as unique identifiers for each ion. The feature matrix was refined by removing ions with missing intensity values (zero intensity) in more than 50% of samples. Internal standards were used for data quality control (QC) to assess reproducibility.

Metabolite identification was performed using Progenesis QI (Waters Corporation, Milford, USA) data processing software, based on multiple public databases including HMDB (http://www.hmdb.ca/), LipidMaps (http://www.lipidmaps.org/), METLIN (https://metlin‐nl.scripps.edu/), PubChem (https://pubchem.ncbi.nlm.nih.gov/), KEGG (https://www.genome.jp/kegg/), and ChEBI (https://www.ebi.ac.uk/chebi/). Additionally, in‐house databases developed by OE Biotech Co., Ltd. (Shanghai) and MAGIGENE (Shenzhen) were also utilized.

The positive and negative data were combined to get a combined data imported into the R ropls package. PCA was carried out to visualize the metabolic alterations among experimental group in organs, while orthogonal partial least square discriminant analysis in the gut microbiota. Differential metabolites were selected on the basis of the combination of a statistically significant threshold of *P* < 0.05 from a two‐tailed Student's t‐test on the normalized peak areas, where metabolites with |Log_2_FoldChange| > 0.26. The metabolite interaction networks were conducted by Cytoscape v3.10.2. And KEGG database was used for functional enrichment analysis.

### Western Blotting

Total protein was extracted from tissue or cell samples using RIPA lysis buffer (Solaribio) supplemented with protease and phosphatase inhibitors (MCE, HY‐K0010). Protein concentrations were determined using the BCA protein assay kit (Thermo Fisher Scientific). Equal amounts of protein (30 µg per lane) were separated by SDS‐PAGE on 4–12% polyacrylamide gels and transferred onto PVDF membranes (Millipore). Membranes were blocked with 5% non‐fat milk for 1 h at room temperature and then incubated overnight at 4 °C with primary antibodies against SGMS1 (proteintech, 19050–1, 1:1000), SMPD1 (proteintech, 14609‐1, 1:1000), SMPD2 (proteintech, 15239‐1, 1:1000), NAMPT (proteintech, 66385‐1‐Ig, 1:1000), and β‐actin (proteintech, 66009‐1‐Ig, 1:1000). After washing, membranes were incubated with HRP‐conjugated secondary antibodies for 1 h at room temperature. Protein bands were visualized using enhanced chemiluminescence (ECL) reagents and imaged with a ChemiDoc Imaging System (Tianneng Co., Shanghai). Band intensities were quantified using ImageJ software, and target protein levels were normalized to β‐actin as internal loading controls.

### NAD^+^, NAM, and NMN Measurement by High Performance Liquid Chromatography‐Mass Spectrometry (HPLC–MS)

Sample preparation and HPLC‐MS test were performed as described previously.^[^ ^88^ ^]^ The LC‐MS quantification was delivered on the Agilent Infinity Lab LC/MSD iQ G6160A. Prepared sample were injected into LC and separated by a ZORBAXSB‐Aq Column (Agilent, 4.6 × 100 mm, 3.5 µm), with 4 °C autosampler temperature and 20 °C column temperature. The following elution program was used: A = aqueous 0.1% formic acid‐water, B = acetonitrile, 0 min 20% A, 0.5 min 20% A, 1.2 min 60% A, 2.5 min 60% A, 2.6 min 20% A, 3 min 20%A, with total run time of 3.0 min and a flow rate of 1.00 mL min^−1^. NAD^+^, NAM, and NMN featured a retention time of 1.09 and 1.06 min respectively. For MS quantification, MS was equipped with electrospray ionization ion source and operates in both positive and negative ion mode. Samples were ionized under high‐flow conditions with curtain gas = 35 psi, collision gas = “high”, cataclastic voltage = 100 V, capillary volt‐age = 3500 V, and dry temperature = 325 °C. Agilent OpenLabData Analysis 2.205.7.2 software was used to do all calculation.

### Targeted Sphingolipidomics by LC‐MS/MS

Cells were counted and transferred into 2 mL centrifuge tubes using 500 µL of methanol–water (v/v = 1:1). Subsequently, 500 µL of chloroform was added, and the samples were subjected to ultrasonic disruption in an ice bath at 1620 W for 10 min using a 6 s on / 4 s off pulse cycle. If an ultrasonic disruptor was not available, mechanical disruption using bead beating (2 min × 2 cycles) was used as an alternative. After disruption, the samples were sonicated for an additional 10 min in an ice bath and incubated at −40 °C for 2–3 h to promote phase separation. The samples were then centrifuged at 13,000 rpm for 10 min at 4 °C, and ≈400 µL of the lower organic phase was carefully collected and evaporated to dryness under vacuum. The dried lipid residues were reconstituted in 200 µL of isopropanol–methanol (v/v = 1:1) containing a mixture of internal standards, vortexed for 1 min, and further extracted by sonication for 3 min. After centrifugation at 13,000 rpm and 4 °C for 10 min, 150 µL of the supernatant was transferred into LC‐MS vials with insert tubes for analysis. QC samples were prepared by pooling equal volumes of all extracted samples to monitor analytical stability and reproducibility throughout the run.

Chromatographic separation was performed using a Waters ACQUITY UPLC HSS T3 column (100 mm × 2.1 mm, 1.8 µm) with a flow rate of 0.4 mL min^−1^ and an injection volume of 5 µL. The mobile phase consisted of solvent A (acetonitrile–water, 60:40, v/v, with 10 mM ammonium formate) and solvent B (acetonitrile–isopropanol, 10:90, v/v, with 10 mM ammonium formate). The elution gradient was programmed as follows: 0 to 1 min, 100% A; 1 to 2 min, linear change to 45% A and 55% B; 2 to 6 min, 40% A and 60% B; 6 to 11 min, 30% A and 70% B; 11 to 12 min, 10% A and 90% B; 12 to 13 min, 100% B; 13 to 14 min, held at 100% B; then returned to 100% A at 14.1 min and re‐equilibrated until 15 min.

Mass spectrometric detection was conducted using an AB Sciex triple quadrupole mass spectrometer operating in multiple reaction monitoring (MRM) mode. The following transitions and optimized parameters were used for targeted sphingolipid analysis: Cer(d18:1/24:1) was monitored at m/z 648.9 → 630.8 with a decluttering potential (DP) of 55 and collision energy (CE) of 19; Cer(d18:1/24:0) at 650.6 → 264.3 with DP 60 and CE 35; SM(d18:1/16:0) at 703.6 → 184.1 with DP 60 and CE 35; SM(d18:1/14:0) at 675.5 → 184.1 with DP 60 and CE 35; SM(d18:1/24:1) at 648.9 → 630.8 with DP 55 and CE 19; SM(d18:1/18:1) at 729.9 → 184.1 with DP 50 and CE 34; and DHCer(d18:0/24:0) at 652.7 → 284.3 with DP 60 and CE 35.

### MitoSOX Red Staining and Intensity Evaluation

After pre‐treatment and oxidative stress, C2C12 cells were loaded into 600 nM MitoSOX Red (Invitrogen/Molecular Probes, M36008) in HBSS for 10 min at 37 °C. Then, cells were washed with warm HBSS buffer. The fluorescent intensity of MitoSOX Red was detected at 480‐nm excitation and 590‐nm emission with 3000‐ms exposure by Olympus IX83. The intensity of signal was evaluated with fold of control in the ImageJ software.

### Integrated Analysis of Behavioral Test, 16S rRNA Sequencing, and Metabolome

Based on results of behavioral tests, relative abundance in 16S rRNA sequencing and variant metabolites in metabolomic analysis, behavioral tests and metabolites were associated to significantly‐changed microbial clustering by Mantel test under Spearman's correlation by R packages LinkET, ggplots2, dplyr and cols4all.

### Behavior‐Metabolome Age Scoring (BMAge) by Z‐Score Standardization

Z‐scores are dimensionless standardized (= normalized) values indicating how many standard deviations a particular observation was below or above the mean of a reference group. In this study, Z‐scores for each individual behavioral and metabolome test were calculated for each animal. The young group was the reference group for calculating the Z‐score of each mouse in the different doses of nicotine. Therefore, in the corresponding test, the Z‐score obtained by each mouse was normalized according to the mean and standard deviation of the young group.

The Z‐score of behavior is divided into 3D behavioral motor ability and 3D behavioral characteristic parameters. Z‐score in the 3D behavioral motor ability was calculated for each animal using the average of normalization of “frame distance” (Z _frame distance_), “movement speed (back)” (Z _movement speed (back)_), “movement energy (back)” (Z _movement energy (back)_), “running” (Z _running_), “Center time ratio” (Z _Center time ratio_) and “ body length ” (Z _body length_) values (against Young group), the 3D behavioral motor ability Z‐score is calculated as (Z _frame distance_ + Z _movement speed (back)_+ Z _movement energy (back)_ + Z _running_ + Z _Center time ratio_ + Z _body length_)/6. Z‐score in the 3D behavioral characteristic parameters was calculated for each animal using the average of normalization of “falling with a right turn” (Z _falling with a right turn_), “rearing” (Z _rearing_), “trotting and exploring” (Z _trotting and exploring_), “walking while sniffing with the head down” (Z _walking while sniffing with the head down_), “walking while sniffing with a left turn” (Z _walking while sniffing with a left turn_), “walking while sniffing with a right turn” (Z _walking while sniffing with a right turn_) and “pausing followed by sniffing” (Z _pausing followed by sniffing_) values (against Young group), Since pausing followed by sniffing varies inversely to the other parameters, the behavior Z score is calculated as (Z _falling with a right turn_ + Z _rearing_ + Z _trotting and exploring_ + Z _walking while sniffing with the head down_ + Z _walking while sniffing with a left turn_ + Z _walking while sniffing with a right turn_ – Z _pausing followed by sniffing_)/7. Z _total_ is calculated as (Z _3D behavioral motor ability_ + Z _3D behavioral characteristic parameters_)/2.

The metabolomic Z‐score was calculated using a normalized average of the values of 0.25Nico and 0.5Nico potential biomarkers (compared to the Young group) to calculate the potential biomarker Z score for each animal. Because the underlying biomarkers change inconsistently, up‐regulated ones are positive and down‐regulated ones are negative. The metabolite Z score was calculated as (Z _up_ – Z _down_/ total number of potential biomarkers).

Eventually, individual test Z‐scores were calculated for each mouse in each cohort. A radar map was drawn based on the Z‐score to evaluate the difference (distance) between different doses of nicotine and the young group and then assess the effect of nicotine. The tendency between the behavioral and metabolomic scores was tested by the simple linear regression.

### Statistical Analysis

Exact values of significance were indicated in all figures. All data were presented as means ± s.e.m. in figures. The experiments were not randomized, and no statistical methods were used to predetermine sample size. The investigators were not blinded during experiments and outcome assessments. Multivariate data were analyzed using one‐way and two‐way ANOVA followed by Tukey's multiple comparisons post hoc tests. Nonparametric data was analyzed by Welch's t‐test. Two‐tailed unpaired t‐test and multiple t‐test with FDR correction analyzed comparisons between two conditions. We used GraphPad Prism version 9.0 and MATLAB 2021a to generate graphs and statistics.

## Conflict of Interest

The authors declare no conflict of interest.

## Author Contributions

S.J. and X.J. contributed equally to this work and are co‐first authors. S.J. conducted experiments, analyzed data, prepared figures, and wrote the original manuscript. X.J. performed data analysis, prepared figures, and contributed to writing the manuscript. R.W. was responsible for animal care during aging and contributed to cell culture. M.S., J.Z., and K.H. performed 3D behavioral data analysis. P.W. (Pei Wang), L.T., and Q.Y. carried out the detection of NAD⁺, NAM, and NMN. Y.F. and W.S. conducted Western blot analyses. X.R. and X.Y. performed targeted LC‐MS/MS sphingolipidomic analysis. P. W. (Ping Wei) and Z.L. contributed to flow cytometry experiments and data analysis. Y.J. contributed to metabolomic pathway analysis. F.H. assisted with adipose tissue distribution measurements. Z.M. assisted with sample collection. C.Y. and L.W. (Liang Wang) supported cell imaging. Q.Z. developed analysis code for quantifying cell fluorescence. Z.Z. conducted organ pathological assessments. G.Z., W.S., F.P., J.W., L.W. (Liping Wang), L.K., and P.K. provided critical analytical tools and assisted with manuscript editing. Z.C. and X.L. conceptualized the project framework and supervised the experiments. X.L. also contributed to data analysis, writing, review, and manuscript editing.

## Supporting information



Supporting Information

Supplemental Tables 1–6

## Data Availability

The data that support the findings of this study are available from the corresponding author upon reasonable request.;
