# Supplementary material for: Nicotine Reprograms Aging‐Related Metabolism and Protects Against Motor Decline in Mice
Source: Adv Sci (Weinh). 2025 Jul 28;12(40):e15311. doi: 10.1002/advs.202415311 (PMC12561400; doi:10.1002/advs.202415311)
Supplement: Supplementary file 1 — Supporting Information [file ADVS-12-e15311-s002.pdf]

# Nicotine reprograms aging-related metabolism and protects against motor decline in mice

*Shuhui Jia [1], Xiaoyuan Jing [1], Ruoxi Wang [1], Mengke Su [2], Pei Wang [3], Yingxin Feng [4], Xiaohu Ren [5], Longfang Tu [3], Ping Wei [6], Zhen Lu [6], Yicong Jia [1], Feng Hong [1], Zhizhun Mo [1], Jiemeng Zou [9], Kang Huang [9], Caiyun Yan [2], Qianhui Zou [1], Liang Wang [10], Guoping Zhong [11], Zhi Zeng [13], Qiuliyang Yu [3], Wen Su [4], Xifei Yang [5], Fan Pan [6], Jianzhi Wang [7], Liping Wang [1], Lijun Kang [12], Paul J. Kenny [8], Zuxin Chen [2 *] and Xin-an Liu [1 *]*

1 Guangdong Provincial Key Laboratory of Brain Connectome and Behavior, Brain Cognition and Brain Disease Institute (BCBDI), Shenzhen-Hong Kong Institute of Brain Science, Shenzhen Institutes of Advanced Technology (SIAT), Chinese Academy of Sciences (CAS), Shenzhen 518055, China

2 Shenzhen Key Laboratory of Drug Addiction, Shenzhen Neher Neural Plasticity Laboratory, Brain Cognition and Brain Disease Institute, Shenzhen-Hong Kong Institute of Brain Science, Shenzhen Institutes of Advanced Technology, Chinese Academy of Science, Shenzhen 518055, China

3 Sino-European Center of Biomedicine and Health, Institute of Biomedicine and Biotechnology, Shenzhen Institutes of Advanced Technology, Chinese Academy of Sciences, Shenzhen 518055, China

4 Department of Pathology, Shenzhen University, Shenzhen 518055, China

5 Shenzhen Key Laboratory of Modern Toxicology, Shenzhen Medical Key Discipline of Health Toxicology, Shenzhen Center for Disease Control and Prevention, Shenzhen 518055, China

6 Centre for Cancer Immunology, Shenzhen Institute of Advanced Technology Chinese Academy of Sciences, Shenzhen 518055, China

7 Department of Pathophysiology, Key Laboratory of Ministry of Education for

Neurological Disorders, School of Basic Medicine, Tongji Medical College, Huazhong University of Science and Technology, Wuhan 430060, China

8 Nash Family Department of Neuroscience, Icahn School of Medicine at Mount Sinai, New York, NY, 10029, USA

9 Shenzhen Bayone Biotech CO., Ltd., Shenzhen 518055, China

10 Research Center for Primate Neuromodulation and Neuroimaging, Shenzhen Key Laboratory for Molecular Imaging, Guangdong Provincial Key Laboratory of Biomedical Optical Imaging Technology, Shenzhen Institutes of Advanced Technology, Chinese Academy of Sciences, Shenzhen 518055, China

11 Institute of Clinical Pharmacology, School of Pharmaceutical Sciences, Sun Yat-sen University, Guangzhou 510080, China

12 Department of Neurology of the Fourth Affiliated Hospital and School of Brain Science and Brain Medicine, NHC and CAMS Key Laboratory of Medical Neurobiology, Zhejiang University School of Medicine, Yiwu 322000, China

13 Department of Pathology, Renmin Hospital of Wuhan University, Wuhan 430060, China

E-mail: xa.liu@siat.ac.cn (X.L.), zx.chen3@siat.ac.cn (Z.C.)

# Supplementary Figures and legends

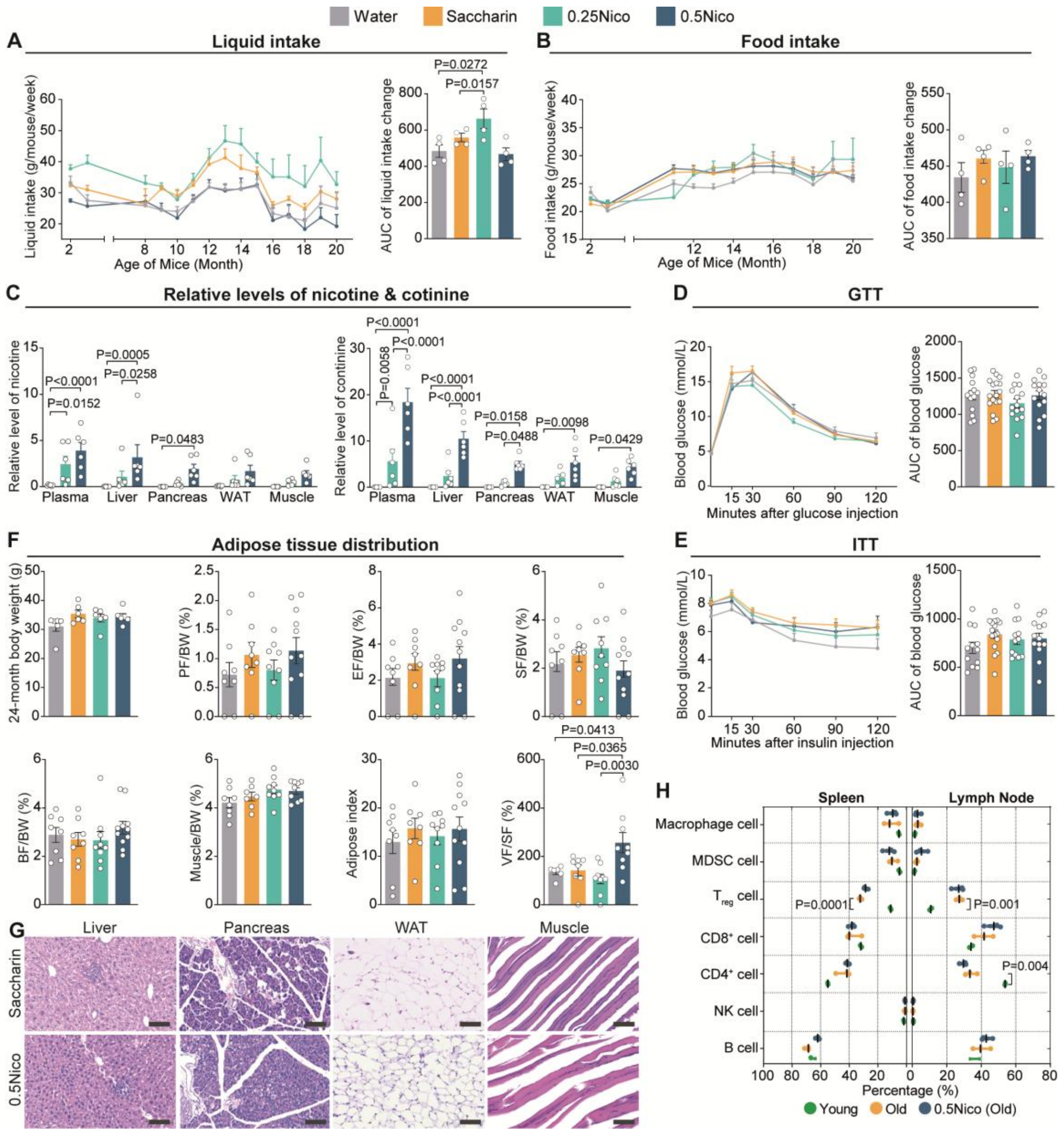


Supplementary Figure 1

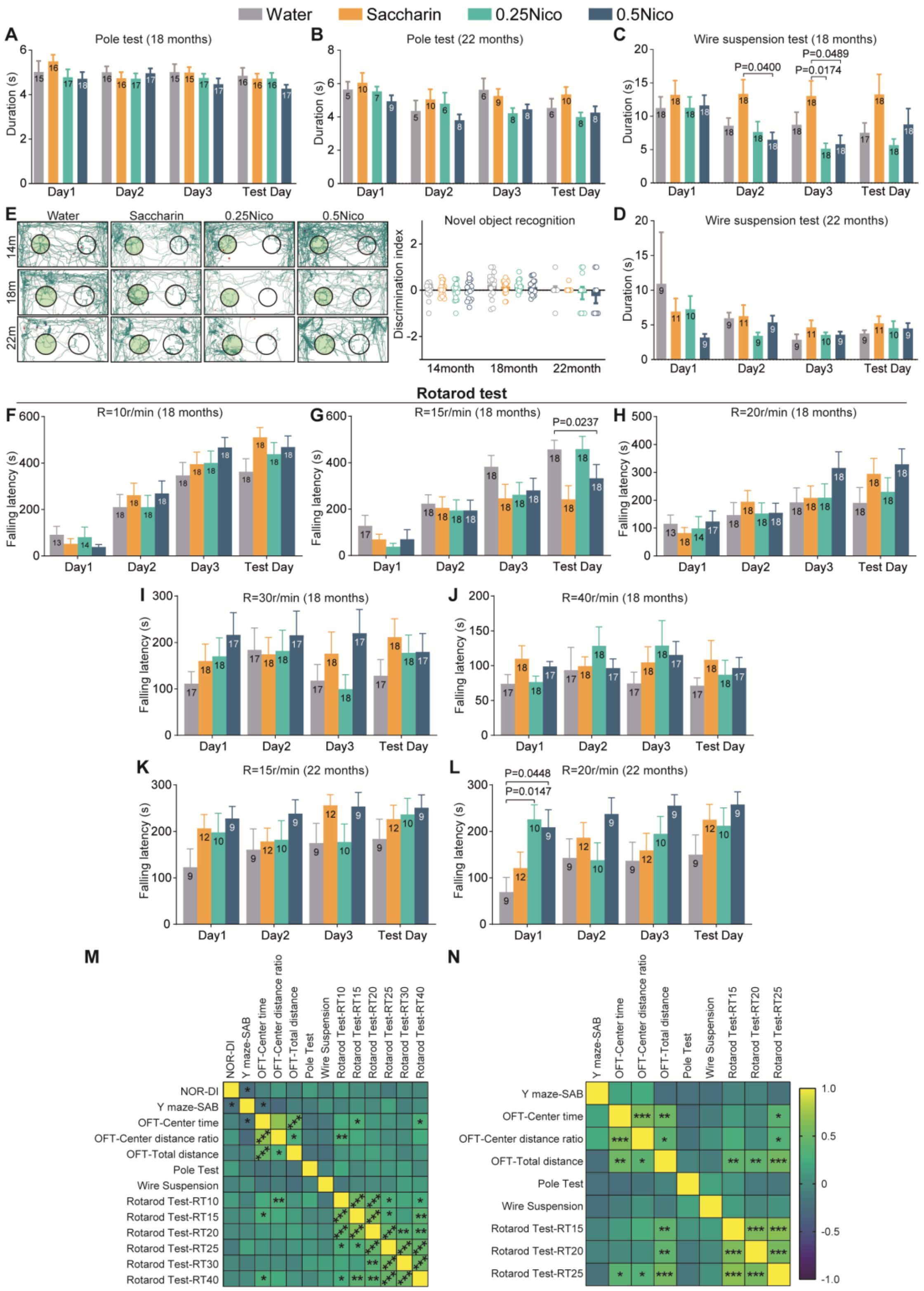


Supplementary Figure 2

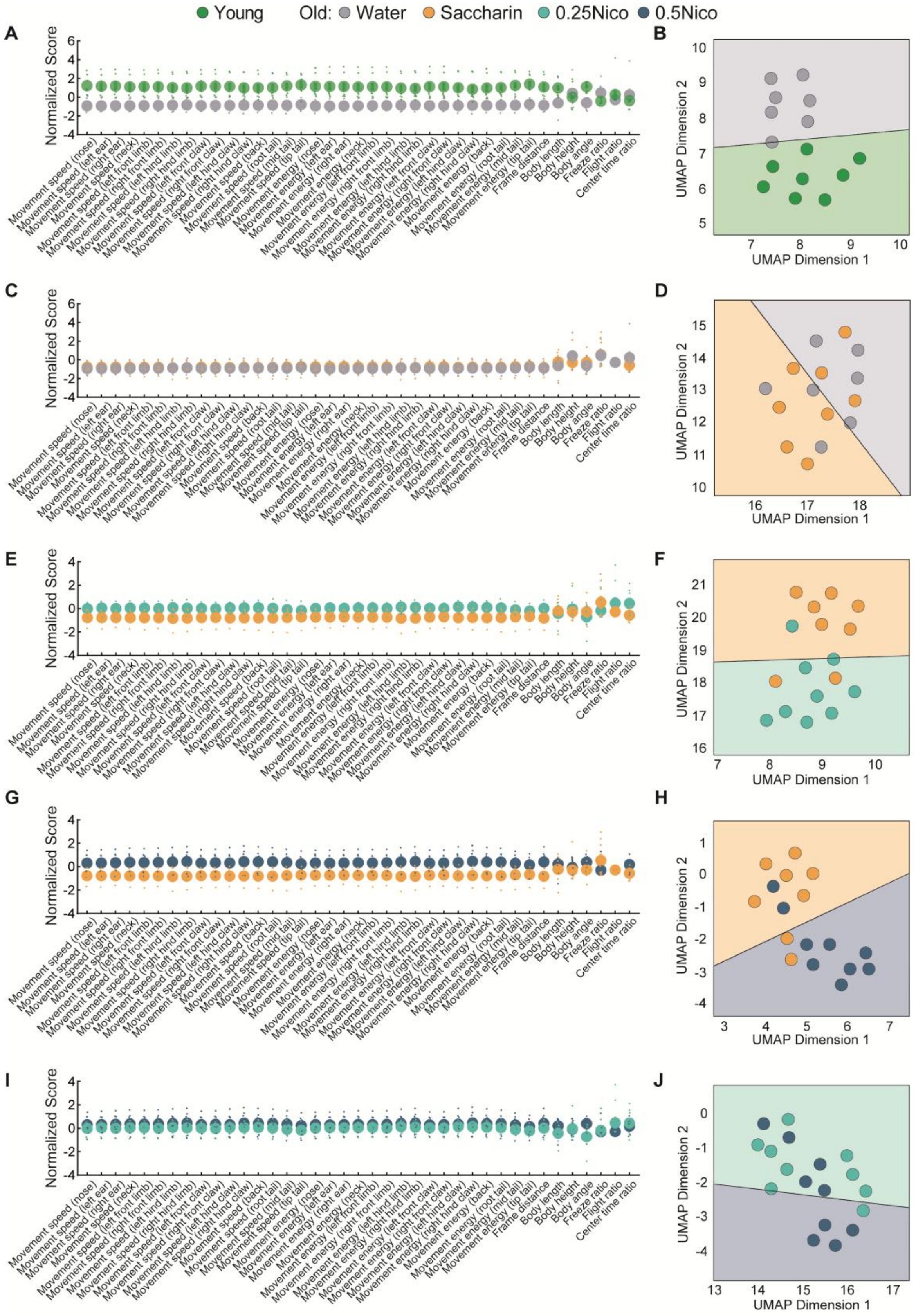


Supplementary Figure 3

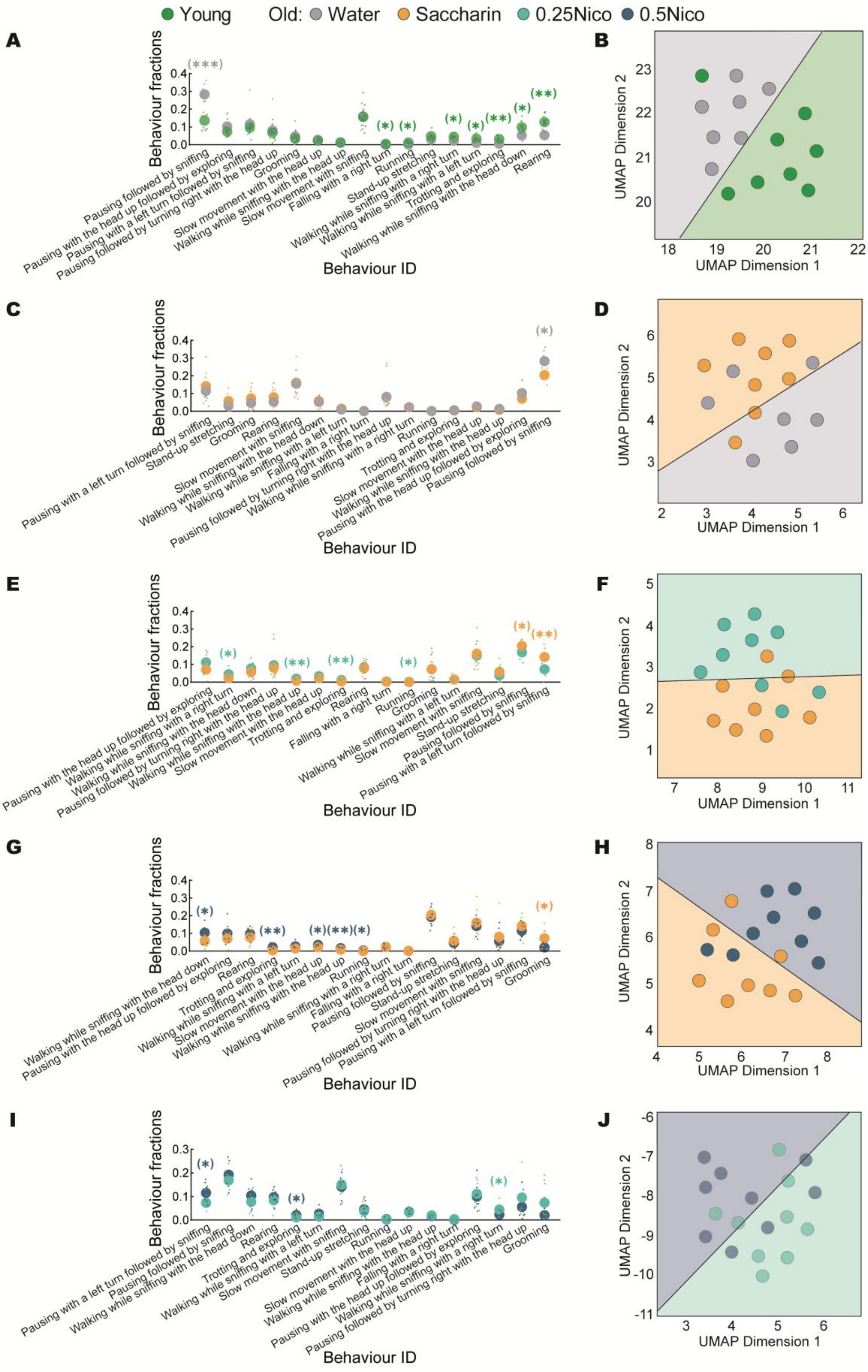


Supplementary Figure 4

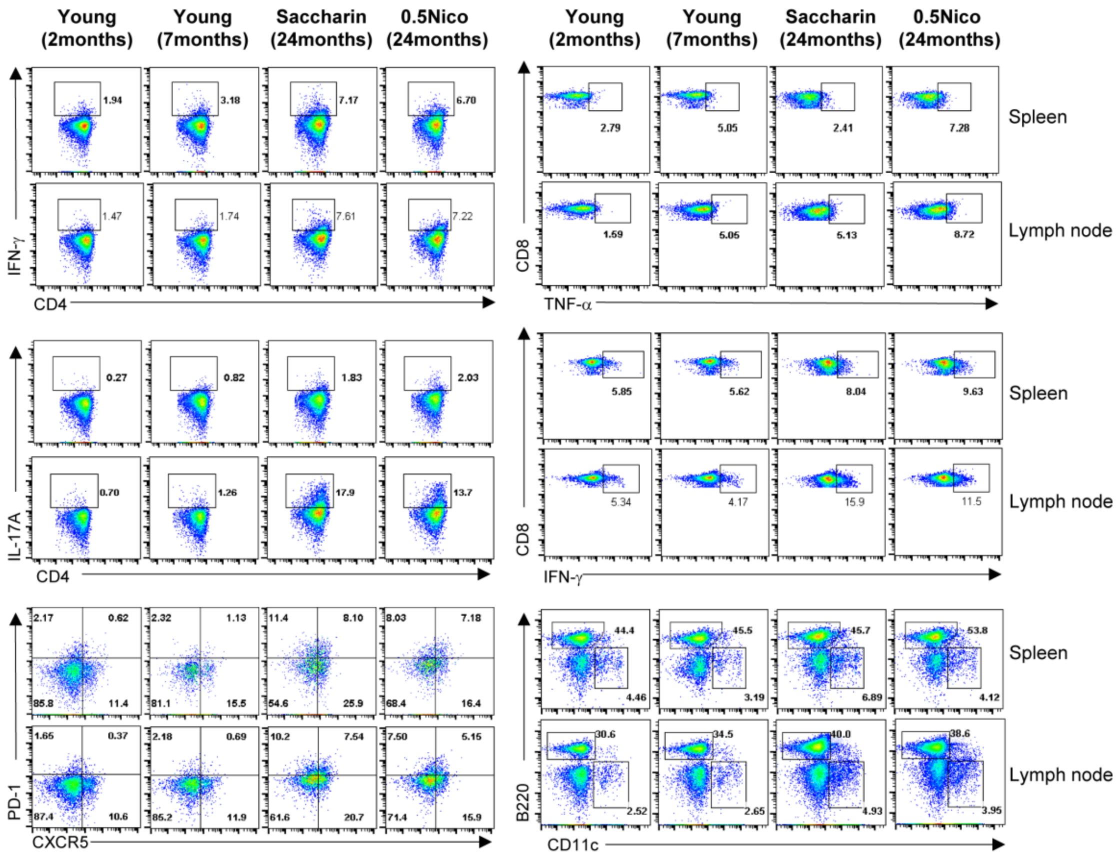


Supplementary Figure 5

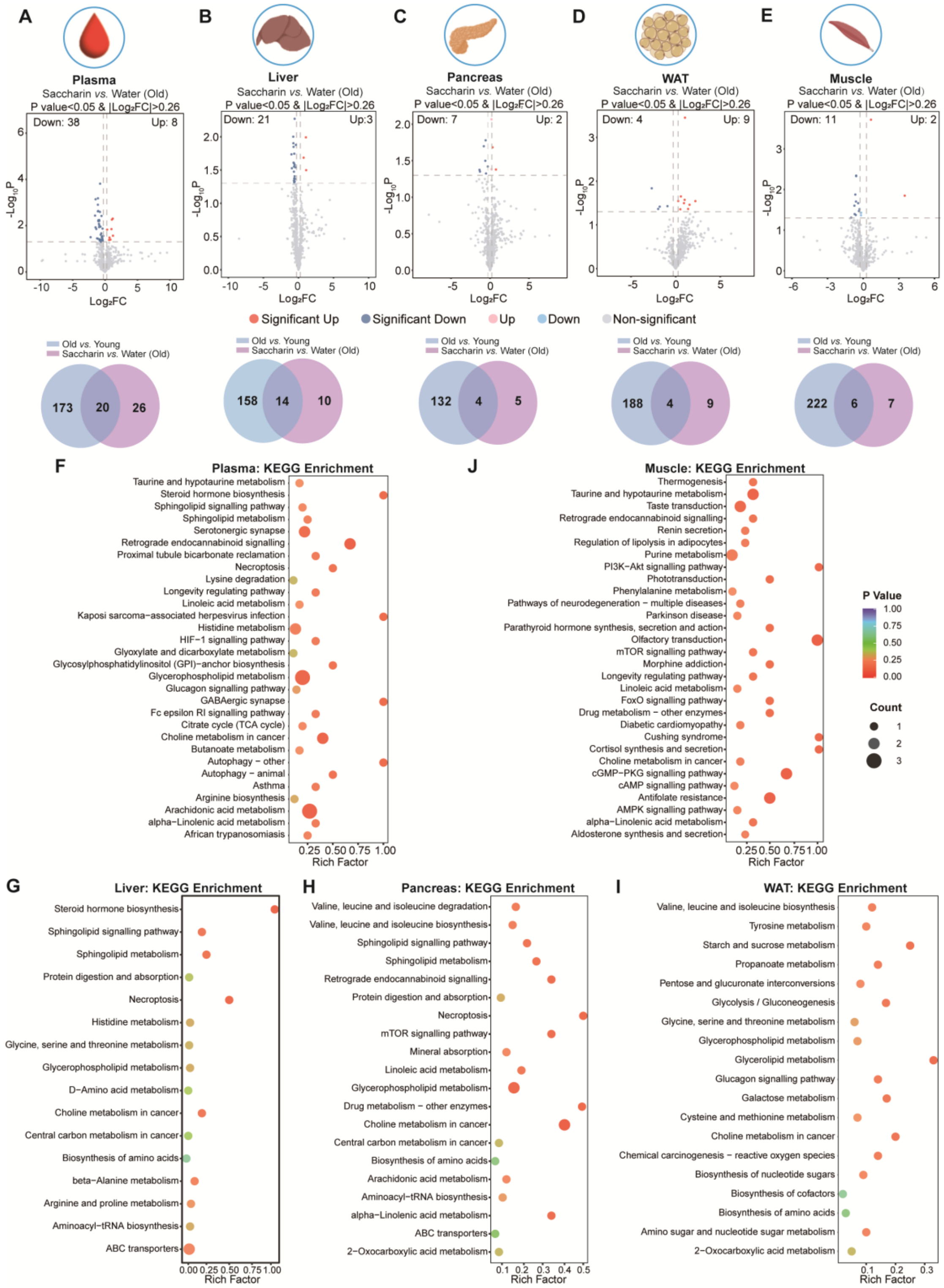


Supplementary Figure 6

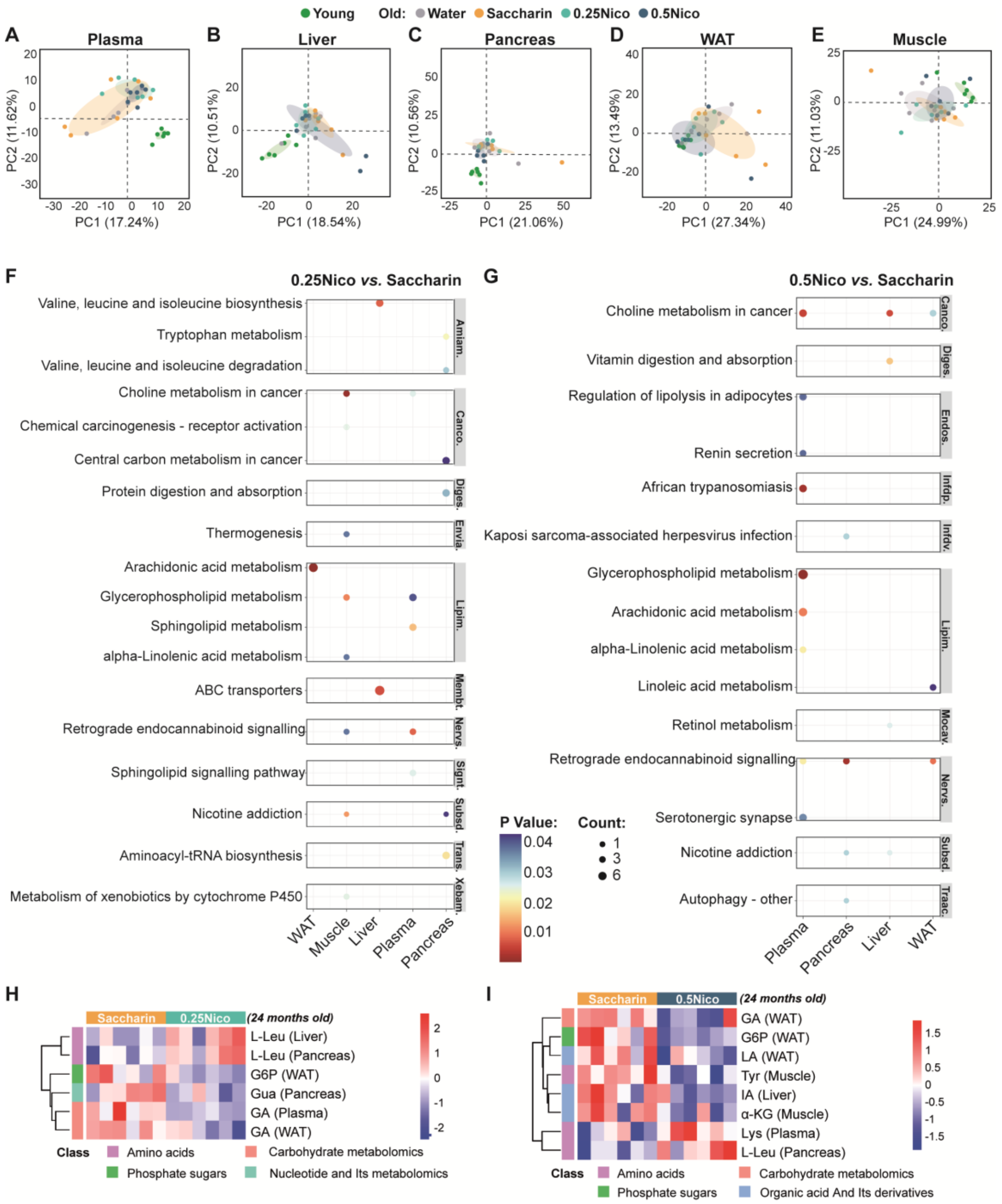


Supplementary Figure 7

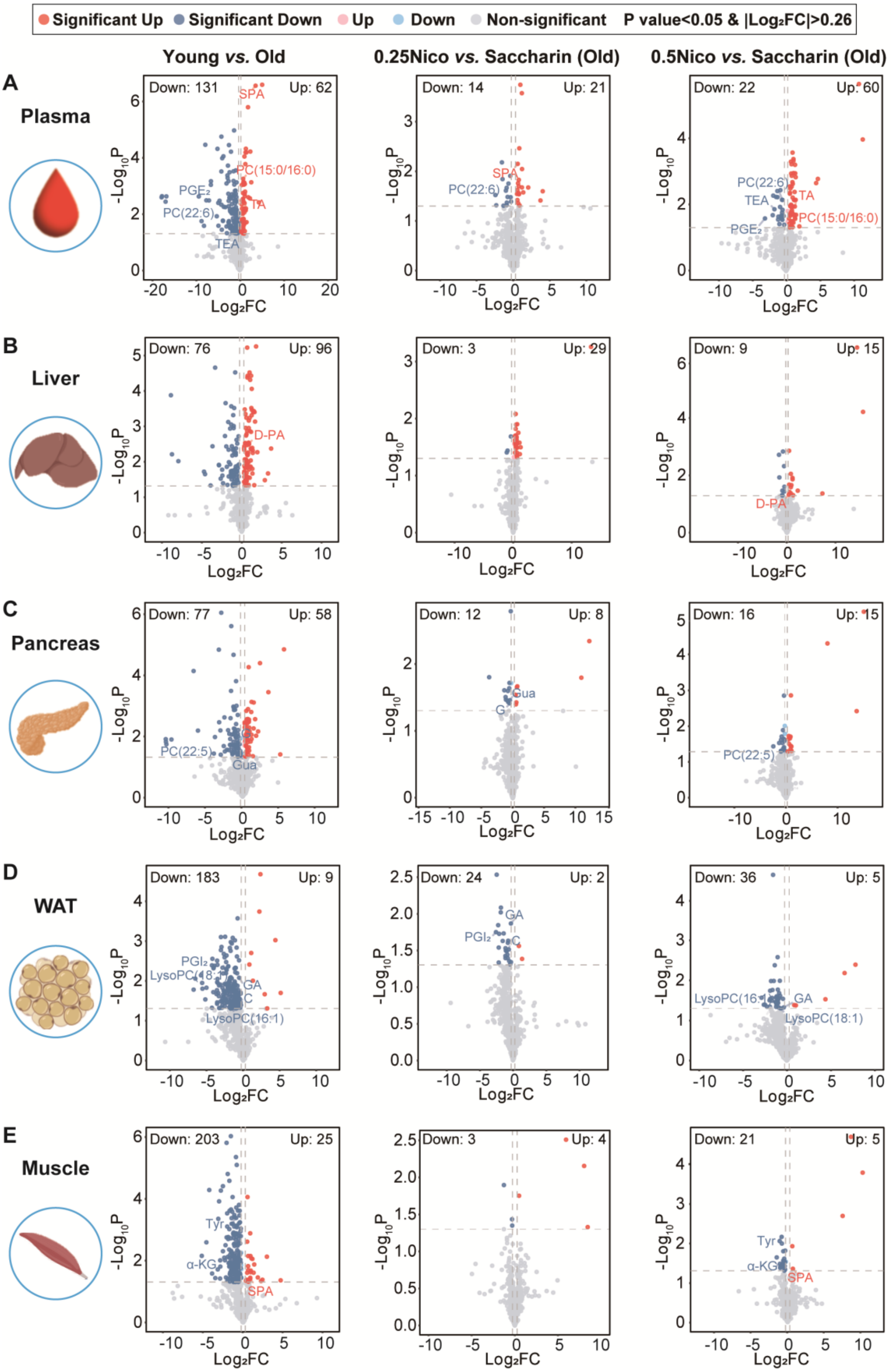


Supplementary Figure 8

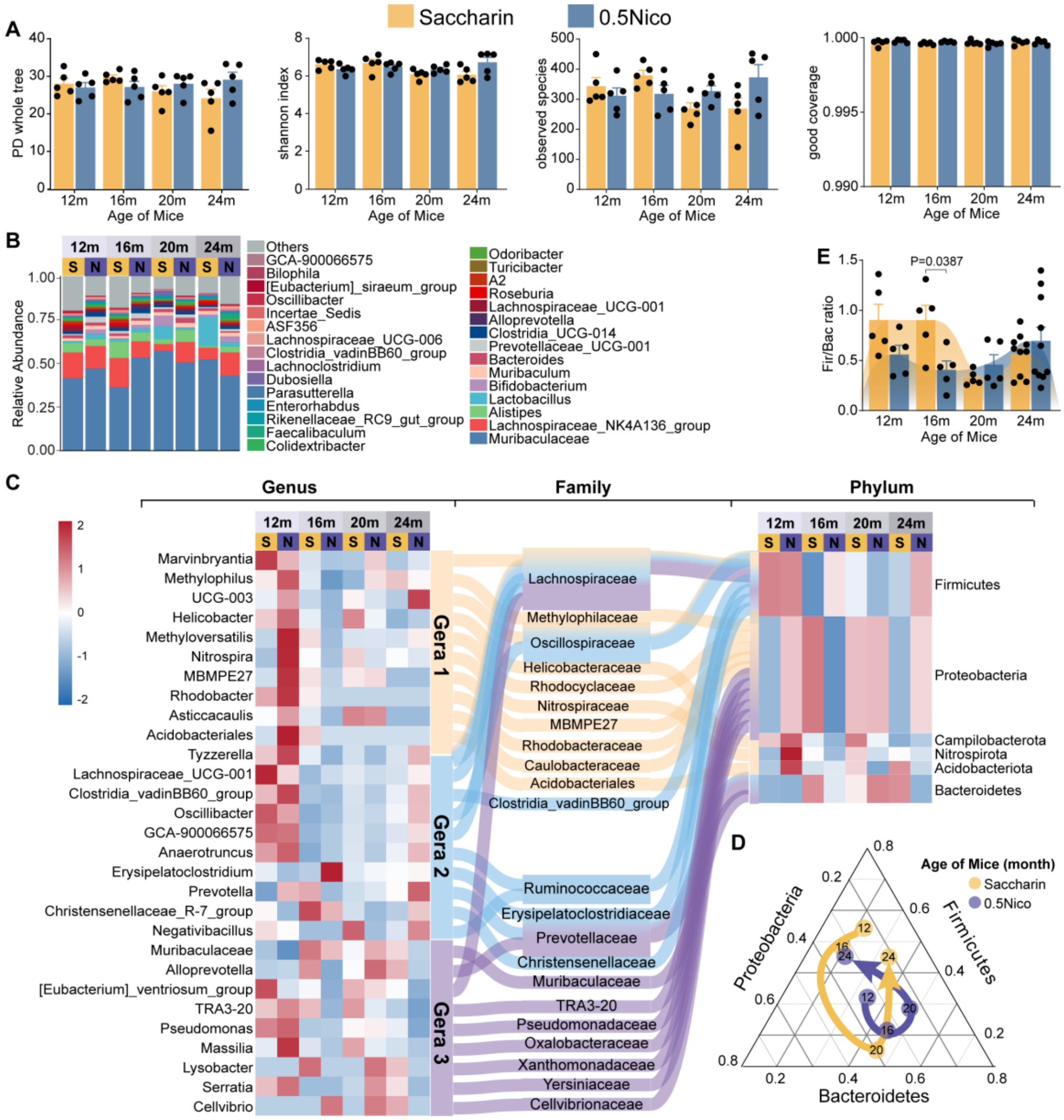


Supplementary Figure 9

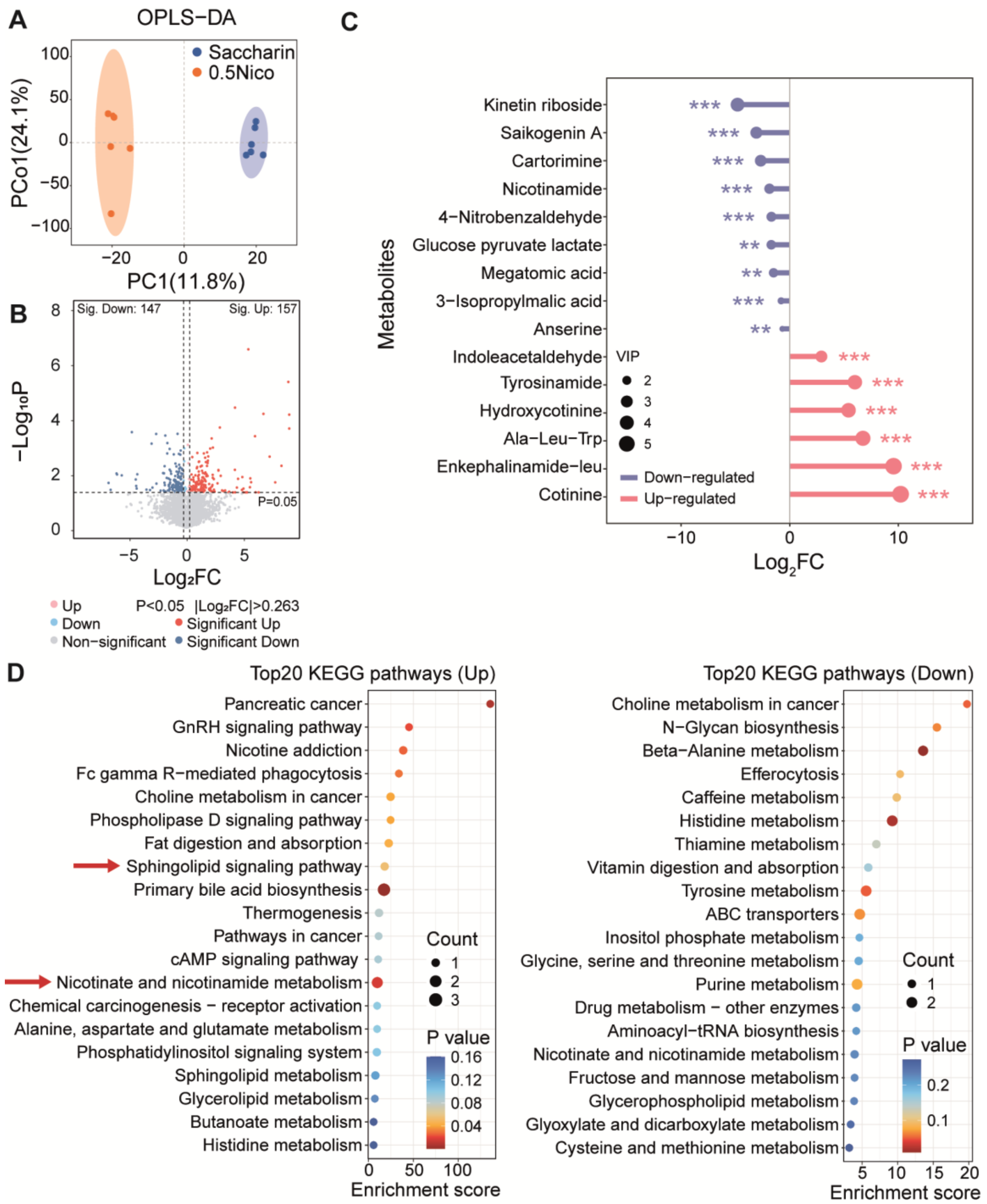


Supplementary Figure 10

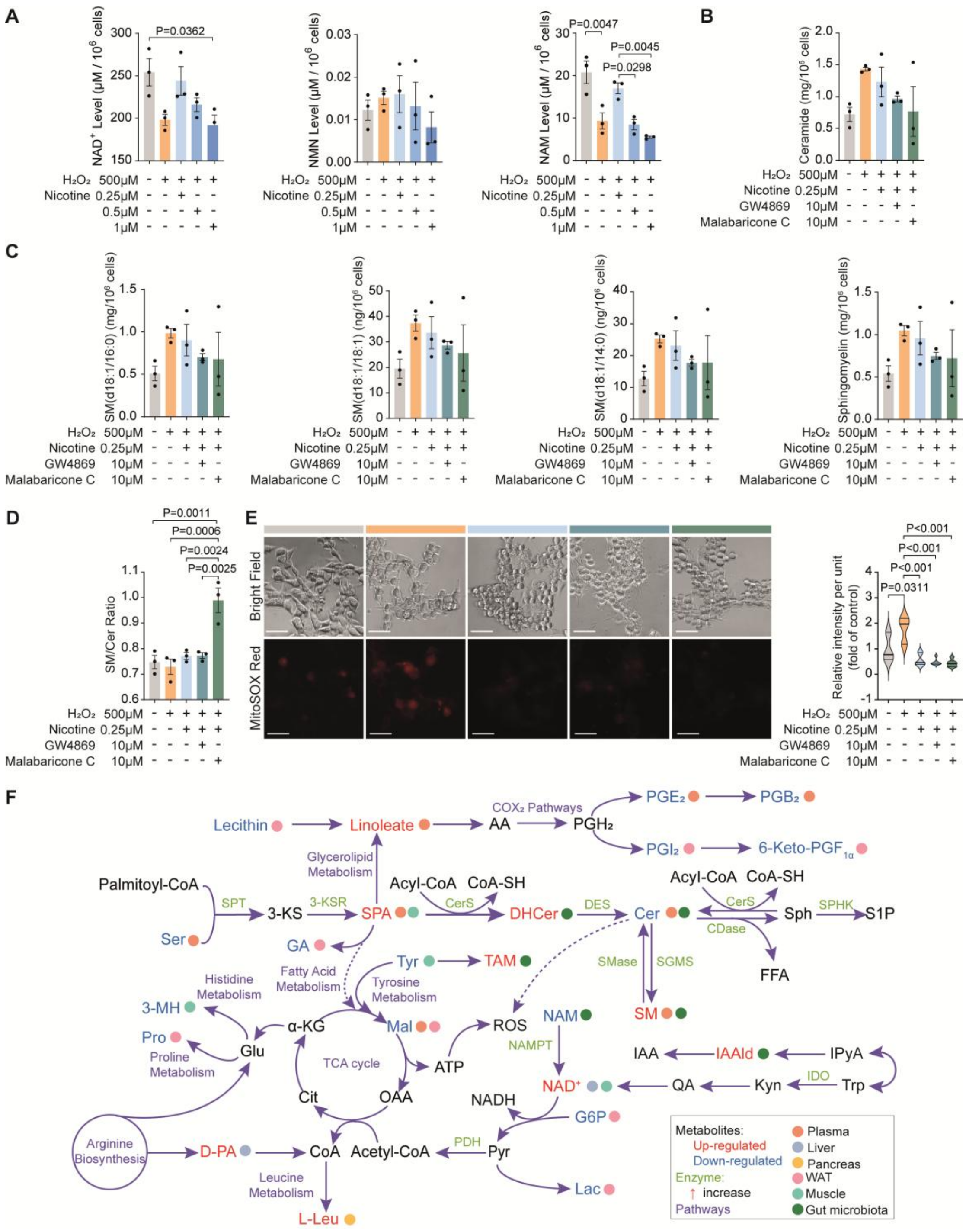


Supplementary Figure 11

**Supplementary Figure 1. Comprehensive assessment of metabolic parameters, organ toxicity, and immune function following oral nicotine exposure.**

A-B, Quantification of liquid (A) and food (B) intake, with AUC analysis for both. C, Relative levels of nicotine and cotinine in major organs, as determined by LC-MS-based metabolomic profiling. D-E, Blood glucose levels and corresponding AUCs from glucose tolerance test (GTT; D) and insulin tolerance test (ITT; E) conducted at 20 months of age. F, Adipose tissue distribution across all experimental groups at 24 months. Body weight is presented, followed by organ-to-body weight ratios derived from fresh tissue measurements, including PF, EF, SF, BF, and representative skeletal muscle. Comparative analyses of the adiposity index and the VF/SF ratio are also shown. G, Representative H&E-stained histological sections of liver, pancreas, WAT, and skeletal muscle from Saccharin- and 0.5Nico-treated mice, imaged at 40 × magnification (scale bar = 100 μm). H, Flow cytometry analysis of immune cell populations in spleen and lymph nodes from Young, Saccharin, and 0.5Nico groups. Two-way ANOVA with Tukey's multiple comparisons test was applied in panels A–B and D–E; one-way ANOVA with Tukey's post hoc test was used in panels C–F and H. Data are presented as mean ± s.e.m., n = 9–20 mice per group. AUC, area under curve; GTT, glucose tolerance test; ITT, insulin tolerance test; SF, subcutaneous fat; PF, perirenal fat; EF, epididymal fat; VF, visceral fat; WAT, white adipose tissue; BW, body weight; MDSC cell, myeloid-derived suppressor cell; $T_{reg}$ cell, regulatory T cell; NK cell, natural killer cell.

**Supplementary Figure 2. Nicotine protects against aging-related motor decline in mice.**

A-B, Performance in the pole test across groups at 18 months (A) and 22 months (B) of age. C-D, Falling latency in the wire suspension test at 18 months (C) and 22 months (D). E, Representative tracking plots and discrimination index from the NOR test across groups. F-J, Falling latency in the constant-speed rotarod test at 18 months of age, with speeds of 10, 15, 20, 30, and 40 r/min. K-L, Falling latency in the constant-speed rotarod test at 22 months, conducted at 15 and 20 r/min. M-N, Correlation heatmaps and representative scatter plots among behavioral test outcomes at 18 months (M) and 22 months (N). Data are presented as mean ± s.e.m. Statistical analyses were performed using two-way ANOVA followed by Tukey's multiple comparisons test. Spearman's correlation was used in (M–N). *p < 0.05, **p < 0.01,

***$p < 0.001$. OFT, open field test; NOR, novel object recognition; DI, discrimination index; SAB, spontaneous alternation behavior.

**Supplementary Figure 3. AI-based quantification of spontaneous behavioral kinematics in mice using a 3D motion-capture system.**

Comparative analyses are presented through paired visualization plots and UMAP with SVM classification for the following experimental groups: (A-B) Young *vs.* Old (Water-treated controls), (C-D) Saccharin *vs.* Water, (E-F) Saccharin *vs.* 0.25 g/L Nicotine (0.25Nico), (G-H) Saccharin *vs.* 0.5 g/L Nicotine (0.5Nico), and (I-J) 0.25 g/L Nicotine (0.25Nico) *vs.* 0.5 g/L Nicotine (0.5Nico). All data represent mean values. UAMP, uniform manifold approximation and projection; SVM, support vector machines.

**Supplementary Figure 4. AI-based quantification of locomotor phenotypes in mice using a 3D motion-capture system.**

Comparative analyses are presented through paired visualization plots and UMAP with SVM classification for the following groups: (A-B) Young *vs.* Old (Water-treated controls), (C-D) Saccharin *vs.* Water, (E-F) Saccharin *vs.* 0.25 g/L Nicotine (0.25Nico), (G-H) Saccharin *vs.* 0.5 g/L Nicotine (0.5Nico), and (I-J) 0.25 g/L Nicotine (0.25Nico) *vs.* 0.5 g/L Nicotine (0.5Nico). Welch's t-test was applied for statistical comparisons in panels A, C, E, G, and I. Data are presented as mean values. *$p < 0.05$, **$p < 0.01$, ***$p < 0.001$. UAMP, uniform manifold approximation and projection; SVM, support vector machines.

**Supplementary Figure 5. Flow cytometry analysis of immune cell populations in spleen and lymph nodes from young control, saccharin-treated, and 0.5** g/L **nicotine-treated (0.5Nico) mice.**

**Supplementary Figure 6. Metabolomic profiling of multi-organ responses to oral saccharin administration in mice.**

A-E, Volcano plots display differential metabolites between Water and Saccharin groups, with Venn

diagrams identifying shared metabolite alterations between Young *vs.* Old and Water *vs.* Saccharin comparisons in (A) plasma, (B) liver, (C) pancreas, (D) WAT, and (E) skeletal muscle. F-J, Bubble plots show KEGG Level 3 pathway enrichment analysis of saccharin-affected differential metabolites in (F) plasma, (G) liver, (H) pancreas, (I) WAT, and (J) muscle. WAT, white adipose tissue.

**Supplementary Figure 7. Metabolomic profile of multiple organs induced by oral nicotine in mice.**

A-E, PCA score plots of metabolites in (A) plasma, (B) liver, (C) pancreas, (D) WAT, and (E) skeletal muscle across experimental groups. F-G, Categorized bubble plots of KEGG Level 3 pathways in (F) 0.25 g/L nicotine (0.25Nico) and (G) 0.5 g/L nicotine (0.5Nico) groups for plasma, liver, pancreas, WAT, and muscle. H-I, Heatmaps of energy metabolism-associated differential metabolites in aged (24-month-old) mice treated with (H) 0.25Nico or (I) 0.5Nico. WAT, white adipose tissue; Amiam, amino acid metabolism; Canco, cancer overview; Diges, digestive system; Envia, environmental adaptation; Lipim, lipid metabolism; Membt, membrane transport; Nervs, nerve system; Subsd, substance dependence; Trans, translation; Xebam, xenobiotics biodegradation and metabolism; Endos, endocrine system; Infdp, infectious disease: parasitic; Infdv, infectious disease: viral; Mocav, metabolism of cofactors and vitamins; Traac, transport and catabolism; L-Leu, L-Leucine; G6P, Glucose 6-phosphate; Gua, Guanosine; GA, Glycolic acid; LA, L-Lactic acid; Tyr, L-Tyrosine; IA, Itaconic acid; α-KG, oxoglutaric acid; Lys, L-Lysine.

**Supplementary Figure 8. Comparative metabolomic analysis of differential metabolites across multiple organs.**

Volcano plots display significantly altered metabolites in (A) plasma, (B) liver, (C) pancreas, (D) WAT, and (E) muscle from the following comparisons: 0.25 g/L nicotine (0.25Nico) *vs.* Saccharin, 0.5 g/L nicotine (0.5Nico) *vs.* Saccharin, and Young *vs.* Old mice. Venn diagrams illustrate overlapping metabolites between Young *vs.* Old and either 0.25Nico *vs.* Saccharin or 0.5Nico *vs.* Saccharin comparisons for each tissue. WAT, white adipose tissue; SPA, Sphinganine; $PGE_2$, Prostaglandin E2; TA, Traumatic acid; PC(22:6), PC(22:6(4Z,7Z,10Z,13Z,16Z,19Z)/18:1(11Z)); TEA, Triethanolamine; D-PA, D-pantothenic acid; PC(22:5), PC(22:5(7Z,10Z,13Z,16Z,19Z)/18:1(11Z)); G, Guanine; Gua,

Guanosine; LysoPC(18:1), LysoPC(18:1(9Z)); GA, Glycolic acid; C, Cytidine; LysoPC(16:1), LysoPC (16:1(9Z)/0:0); $PGI_2$, Prostaglandin I2; Tyr, L-Tyrosine; α-KG, oxoglutaric acid.

**Supplementary Figure 9. Longitudinal profiling of gut microbiota dynamics in aging mice treated with saccharin or nicotine.**

A, Temporal changes in alpha diversity indices (PD whole tree, Shannon index, observed species, and Good's coverage) across experimental groups at 12, 16, 20, and 24 months of age. B, Stacked bar plots showing taxonomic composition in saccharin- and 0.5 g/L nicotine (0.5Nico)-treated groups at each timepoint. C, Heatmap visualization of differentially abundant microbial genera (with corresponding family and phylum classifications) showing significant temporal patterns in saccharin and 0.5Nico groups. D, Ternary plots illustrating phylum-level community structure across age groups. E, Comparative analysis of the Fir/Bac ratio between treatment groups during aging. Multiple t-test with FDR correction was used in A and E. Data represent mean ± s.e.m, n=5. *p < 0.05. Fir, Firmicutes; Bac, Bacteroidetes; PD, phylogenetic diversity; FDR, false discovery rate.

**Supplementary Figure 10. Metabolomic profiling of gut microbiota in 24-month-old mice.**

A, OPLS-DA score plot demonstrating distinct clustering of gut microbial metabolites across experimental groups. B, Volcano plot identifying significantly differentially abundant metabolites between 0.5 g/L nicotine (0.5Nico)-treated and saccharin control groups. C, Lollipop plot of significantly regulated microbial metabolites (multiple t-tests with FDR correction, **p < 0.01, ***p < 0.001; n = 5 per group). D-E, KEGG pathway enrichment analysis showing the top 20 significantly upregulated (D) and downregulated (E) metabolic pathways in 0.5Nico *vs.* saccharin groups.

**Supplementary Figure 11. Sphingolipid metabolism and energy regulation in skeletal muscle and C2C12 cells following nicotine exposure.**

A, HPLC quantification of $NAD^+$ metabolites ($NAD^+$, NMN, NAM) in C2C12 cells following $H_2O_2$-induced oxidative stress with or without nicotine pre-treatment. B-C, LC/MS-based sphingolipid profiling showing Cer levels (B), Specific sphingomyelins [SM(d18:1/16:0), SM(d18:1/18:1),

SM(d18:1/14:0)] and total sphingomyelin content (C) in C2C12 cells exposed to $H_2O_2$-induced oxidative stress, with or without pre-treatment using nicotine and inhibitors of sphingomyelin synthase (SMS) or neutral sphingomyelinase (nSMase). D, SM/Cer ratio in C2C12 cells under oxidative stress following pre-treatments with or without nicotine and sphingolipid pathway inhibitors, assessed by LC-MS. E, Confocal microscopy of mitochondrial ROS (MitoSOX Red stain) in oxidative stress-challenged C2C12 cells with indicated treatments (scale bar = 50 μm). F, Schematic overview of metabolic pathways significantly altered across multiple organs and fecal samples in 24-month-old mice following lifelong nicotine exposure. Data are presented as mean ± s.e.m; n = 3 independent experiments per group. One-way ANOVA followed by Tukey's multiple comparisons test was used for statistical analysis. AA, arachidonic acid; $PGH_2$, prostaglandin H2; $PGE_2$, prostaglandin E2; $PGB_2$, prostaglandin B2; $PGI_2$, Prostaglandin I2; 6-keto-$PGF_{1\alpha}$, 6-ketoprostaglandin $F_{1\alpha}$; Ser, serine; SPT, serine palmitoyl-transferase; 3-KS, 3-keto-spinganine; SPA, sphinganine; CerS, ceramide synthase; DHCer, dihydroceramide; DES, dihydroceramide synthase; Cer, ceramide; CDase, ceramidase; FFA, free fatty acid; Sph, sphingosine; SPHK, sphingosine kinase; S1P, sphingosine-1-phosphate; GA, glycolic acid; 3-MH, 3-methylhistidine; Pro, L−proline; Glu, glutamine; α-KG, oxoglutaric acid; Tyr, L-Tyrosine; TAM, tyrosinamide; Mal, malate; Cit, citrate; OAA, Oxaloacetic Acid; ATP, adenosine triphosphate; ROS, reactive oxygen species; NAM, nicotinamide; SM, sphingomyelin; SGMS, sphingomyelin synthase; SMase, sphingomyelinase; $NAD^+$, nicotinamide adenine dinucleotide; NADH, nicotinamide adenine dinucleotide; G6P, glucose 6-phosphate; Lac, lactate; Pyr, Pyruvate; PDH, Pyruvate dehydrogenase; CoA, coenzyme A; L-Leu, L-Leucine; D-PA, D-pantothenic acid; IAA, indole-3-acetic acid; IAAld, indole-3-acetaldehyde; IPyA, indole-3-Pyruvic acid; Trp, Tryptophan; Kyn, kynurenine; IDO, indoleamine 2,3-dioxygenase; QA, quinolinic acid; 3-KSR, 3-keto-dihydrosphingosine reductase.
